# Supplementary material for: Synthesizing theories of human language with Bayesian program induction
Source: Nat Commun. 2022 Aug 30;13:5024. doi: 10.1038/s41467-022-32012-w (PMC9427767; doi:10.1038/s41467-022-32012-w)
Supplement: Supplementary file 1 — Supplementary Information [file 41467_2022_32012_MOESM1_ESM.pdf]

Supplementary Information for: Synthesizing Theories of Human Language with Bayesian Program Induction

Kevin Ellis*, Adam Albright, Armando Solar-Lezama, Joshua B. Tenenbaum, Timothy J. O'Donnell
*: To whom correspondence should be addressed. E-mail: kellis@cornell.edu

Contents

S1 Supplemental Discussion: Linguistic Formalism **1**

S2 Supplemental Figures **2**
S2.1 Textbook problems 2
S2.2 Artificial grammar learning 2
S2.3 Learned Fragment-Grammar meta-model 2

S3 Supplemental Methods **9**
S3.1 Input data format 9
S3.2 Counter-Example Guided Inductive Synthesis 9
S3.3 Incremental synthesis of grammars 9
S3.4 Bayesian prior over grammars 11
S3.5 Ablation studies 12

S4 Full set of problems and model outputs **12**

S1 Supplemental Discussion: Linguistic Formalism

Surface and underlying forms. The final pronounced form of a word is referred to as its *surface form*, conventionally written between brackets, while intermediate forms such as entries in the lexicon are called *underlying forms* and are written between slashes. For example, the English past tense of the word walk (i.e. "walked") has the surface form [wɔkt], but the underlying form /wɔkd/, which is built from the underlying form of the past tense (i.e. /d/) and the underlying form of the stem for walk (i.e. /wɔk/). In the main text we have adopted a simplified presentation where all (sequences of) phonemes are written between slashes.

Morphosyntax. In modern linguistic theories, the mapping between form and meaning is assumed to be mediated by a central component called *(morpho)syntax* which contains information about the category of constituents and how they combine. Thus, each linguistic constituent is described by a form-category-meaning triple $\langle f, c, m\rangle$. In the present work, these components consist of:

1. **Form**: A specification of the sound structure of the constituent. In this work, we will use sequences of phonemes described as phonetic feature vectors. For example, the English (EN) past tense verb form *walked* is represented as [wɔkt]. This complex constituent consists of two smaller pieces, the past tense suffix which is represented underlyingly as /d/ and the stem /wɔk/.

2. **Morphosyntactic Category**: A specification of the category of the constituent and how it combines with other constituents. The category of *walked* is verb `V`. The past tense marker /d/ has category `V\V` meaning that it must attach to a verb stem on it's left to produce a verb. Analogously, a morpheme that is prefixed (e.g., 're-', as in 'reanalyze', 'redo', ...) would have the morphosyntactic category `V/V`. In the main text, we used the more intuitive, less formal markers `pfx`, `stem`, `sfx` for `V/V`, `V`, `V\V`, respectively.

3. **Meaning**: A specification of the meaning. In this work, we assume meanings are just sets of atomic meaning features. So the meaning of *walked* is [**stem**:WALK;**tense**:PAST]. The meaning of the past tense marker /d/ is [**tense**:PAST] and the meaning of the stem *walk* is [**stem**:WALK].

Thus, *walked* will be represented as $\langle[\text{wɔkt}], \texttt{V}, [\textbf{stem}{:}\text{WALK};\textbf{tense}{:}\text{PAST}]\rangle$. A grammar **G** can be thought of as a joint distribution on the set X of form-category-meaning triples: $\langle f, c, m\rangle \in X$ and is specified by four components $\mathbf{G} = \langle \mathbf{L}, \mathbb{S}, (\!|\cdot|\!), [\![\cdot]\!]\rangle$.[1] First, there is a stored collection of primitive or atomic units known as the lexicon **L**. Each lexical item is also a form-category-meaning triple. Lexical items are assembled into more complex structures using an inventory of structure building operations $\mathbb{S}$. In our model, $\mathbb{S}$ corresponds to concatenation of morphemes. Finally, assembled morphosyntactic structures are mapped to surface sound and meaning representations by a pair of functions called *interface mappings* which take syntactic objects to sound structure $(\!|\cdot|\!)$ (*phonology*) and meaning structure $[\![\cdot]\!]$ (*semantics*) respectively.

For example, in English, the stem for the verb *walk* is $\langle/\text{wɔk}/, \texttt{V}, [\textbf{stem}{:}\text{WALK}]\rangle \in \mathbf{L}_{\text{EN}}$. In our model, we adopt a single structure-building operation $\sigma(\cdot,\cdot)$ that operates on pairs of constituents concatenating their form parts, algebraically canceling their category parts, and performing unification on their meaning. For example, in English the past tense is marked regularly by the lexical item $\langle/\text{d}/, \texttt{V}\backslash\texttt{V}, [\textbf{tense}{:}\text{PAST}]\rangle$. So the past tense form *walked* can be constructed as

$$\sigma(\langle/\text{wɔk}/, \texttt{V}, [\textbf{stem}{:}\text{WALK}]\rangle, \langle/\text{d}/, \texttt{V}\backslash\texttt{V}, [\textbf{tense}{:}\text{PAST}]\rangle) = \langle[\text{wɔkd}], \texttt{V}, [\textbf{stem}{:}\text{WALK};\ \textbf{tense}{:}\text{PAST}]\rangle$$

Note that the output of σ represents the *underlying form* /wɔkd/—on the surface the final /d/ is devoiced in the context of the voiceless /k/; that is, it is pronounced [wɔkt]. The set of all possible underlying forms is simply the closure of the lexicon **L** under σ. We write $U_{\mathbf{L}}$ for the set of all underlying forms in a language with lexicon **L**—that is, the set of all structures derivable from **L** using σ.

The interface function $(\!|\cdot|\!)$ is a set of ordered transduction (SPE-style) rules which map underlying to surface forms. In the present work, we leave $[\![\cdot]\!]$ as the identity function.

Using the formalism introduced above, the theory-induction objective corresponds to finding a set of phonological transduction rules $(\!|\cdot|\!)$, semantic transduction rule $[\![\cdot]\!]$ and lexicon **L** maximizing

$$\left[\prod_{x\in\mathbf{X}} \mathbb{1}[(\exists u) \mid u \in U_{\mathbf{L}} \wedge (\!|u|\!) = x.f \wedge [\![u]\!] = x.m]\right] P(\mathbf{G}|\text{UG})$$

which is equivalent to Eq. 1 in the main paper.

S2 Supplemental Figures

S2.1 Textbook problems

Figure S1-S3 illustrates grammars learned for additional textbook problems.

S2.2 Artificial grammar learning

Figure S4 shows learning curves for the model on artificial grammar learning experiments.

S2.3 Learned Fragment-Grammar meta-model

Fig. S5 shows our basic context free grammar over SPE-style rewrites, which defines a space of possible programs for modelling phonological rules. In learning a meta-model, we build on top of this hand-coded basis. Specifically, we learn by adding more context-free production rules to this basic grammar. This works by learning additional commonly occurring fragments of rules (Fig. S6), which biases the model toward reusing those fragments.

[1] In the main paper we factor grammars into rules **T** and lexicon, **L**. This section combines these two factors into a single tuple, the grammar **G**, and also incorporates morphosyntactic category information in the observed data, which we elided from the main text. Our system assumes every observed (form, meaning) pair has the same morphosyntactic category.

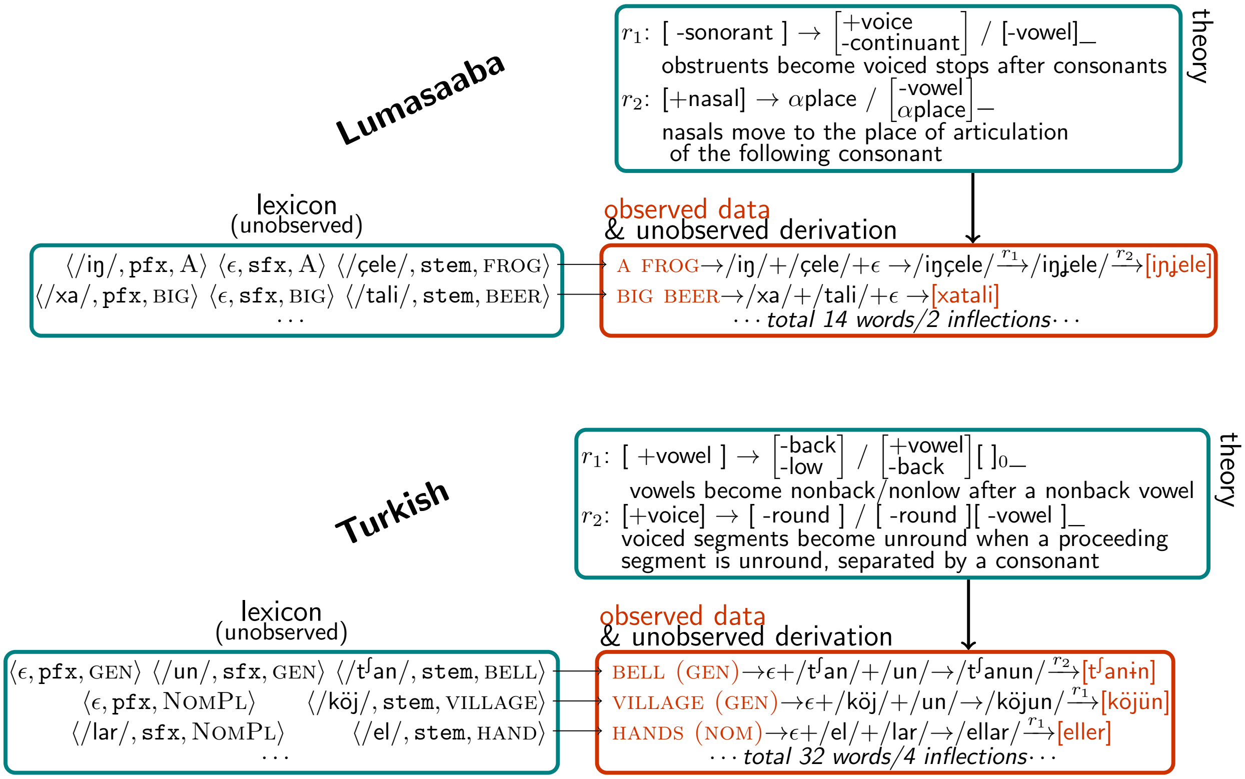

Figure S1: Given dataset, highlighted in orange, system jointly infers both language-specific phonological rules ("theory" box, labeled r_1, r_2, etc.) and dataset-specific lexicon, which include both stems and affixes for each inflection. Together the theory and lexicon explain the orange data via a derivation where the morphology output (prefix+stem+suffix) is transformed according to the ordered rules. The symbol ϵ means the empty string.

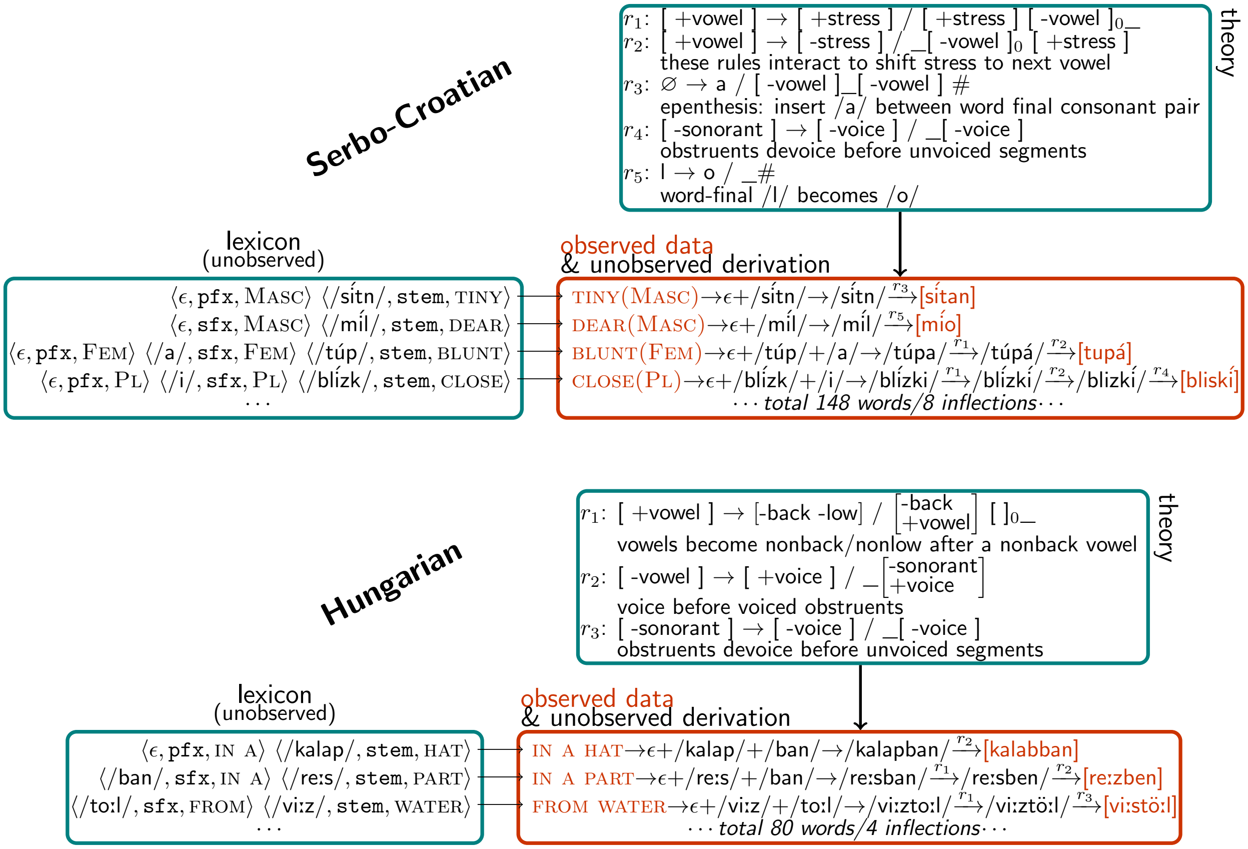

Figure S2: Given dataset, highlighted in orange, system jointly infers both language-specific phonological rules ("theory" box, labeled r_1, r_2, etc.) and dataset-specific lexicon, which include both stems and affixes for each inflection. Together the theory and lexicon explain the orange data via a derivation where the morphology output (prefix+stem+suffix) is transformed according to the ordered rules. The symbol ϵ means the empty string.

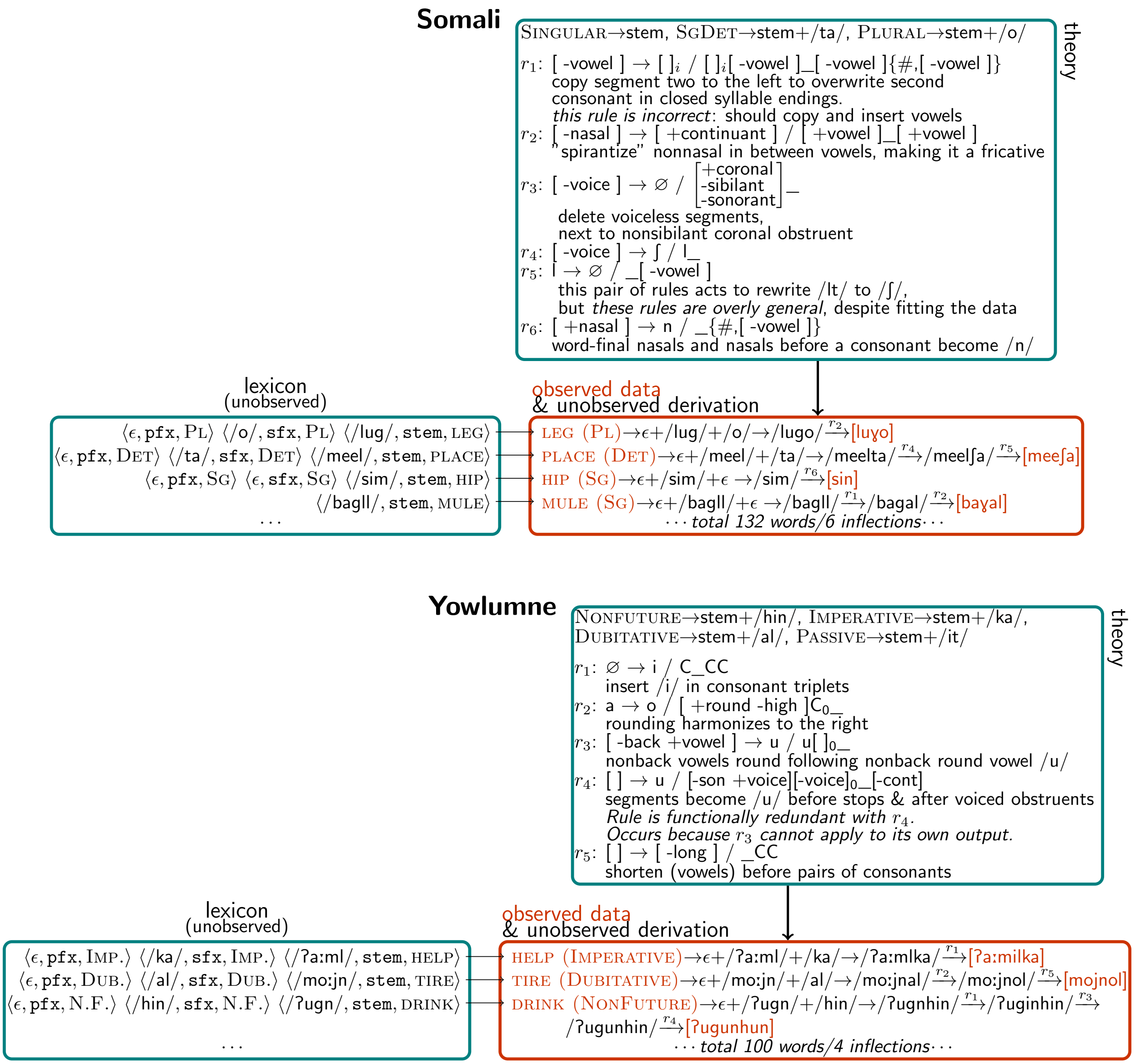

Figure S3: Example failure modes for our system; illustration is analogous to Fig. S1-S2. Somali rule system fails to explain 20% of the textbook problem, and many of the individual rules are implausible upon inspection, such as the first copying rule r_1 — see the bottom of derivation of the singular form of "mule." Other rules are essentially correct, such as the spirantization process implemented by rule r_2, or the neutralization process in r_6. Yowlumne rule system contains a redundant vowel rounding process, r_4, which acts in concert with r_3 to repeatedly harmonize segments to /u/; see illustration of the derivation of the non-future form of DRINK (bottom of observed data/unobserved derivation box). ("Yowlumne" was formerly known as "Yawelmani" [1].)

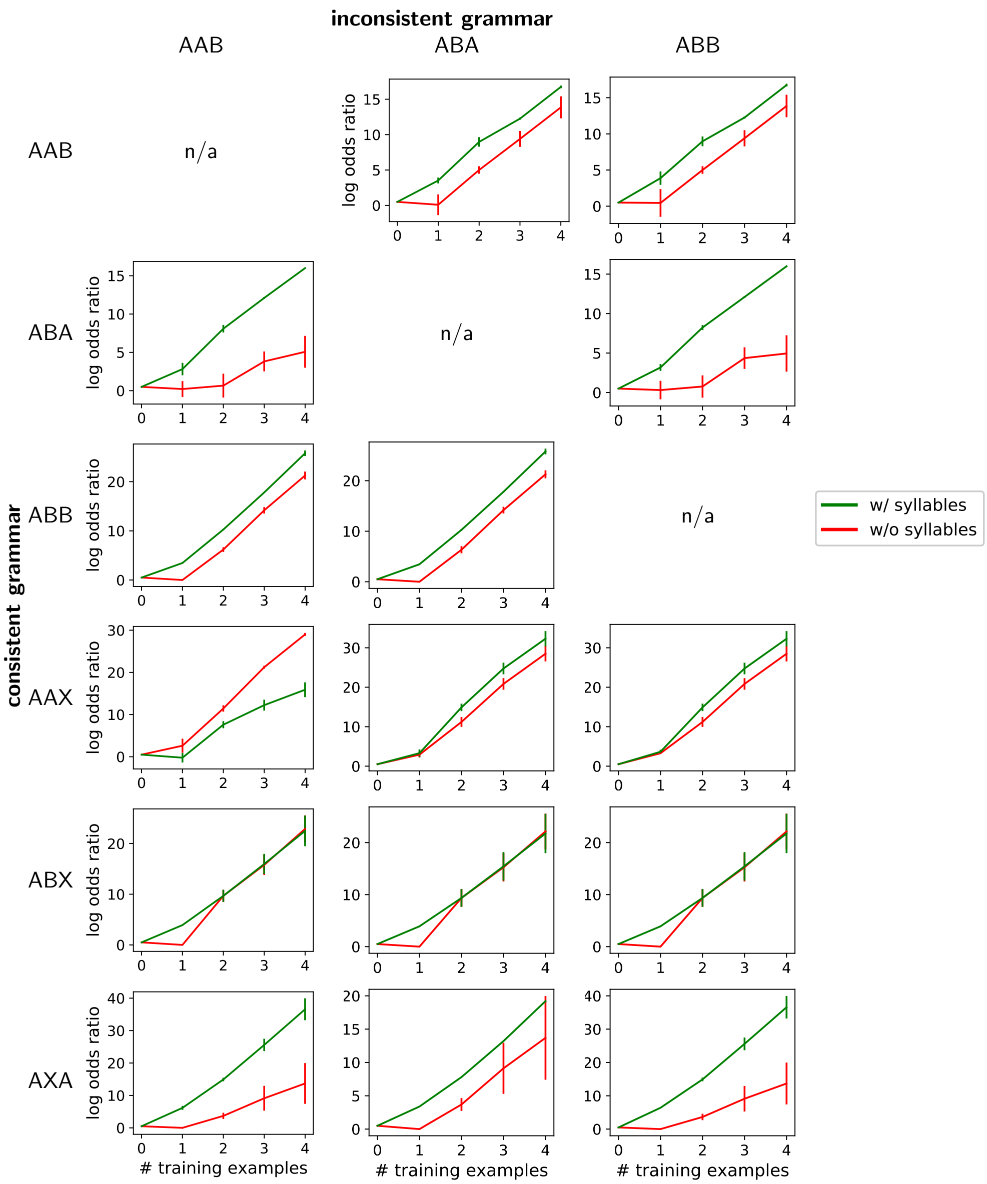

Figure S4: Learning curves artificial for grammar learning (compare with Fig. 6). The x-axis of each plot varies the number of training words, each drawn from the 'consistent' grammar. The y-axis of each plot compares the likelihood of test words from the consistent and inconsistent grammars according to the log odds ratio $\log P(\text{consistent}|\text{train})/P(\text{inconsistent}|\text{train})$. Values greater than 0 indicate successful discrimination between the consistent and inconsistent grammars. Curves show mean and standard deviation over $n = 15$ random test word pairs conforming to the (in)consistent grammars. Green: w/ syllabic representation. Red: w/o syllabic representation. Source data are provided as a Source Data file.

	Template for a single rule	
Rule ::=	Focus→Change / Trigger _ Trigger	
	Focus of rule, and what it can change into	
Focus ::=	∅	*insertion rule*
Focus ::=	FeatureMatrix	
Change ::=	∅	*deletion rule*
Change ::=	$\mathbb{Z}$	*copying rule: an integer*
Change ::=	αplace	*copy place from α, an integer*
Change ::=	FeatureMatrix	
	Every triggering environment with at most two feature matrices	
Trigger::=		*empty conditioning (triggering) environment*
Trigger::=	FeatureMatrix	*a single feature matrix to the side of focus*
Trigger::=	FeatureMatrix_0 FeatureMatrix	*zero or more repeats of a feature matrix*
Trigger::=	FeatureMatrix FeatureMatrix	
Trigger::=	#	*end-of-string*
Trigger::=	FeatureMatrix #	
Trigger::=	FeatureMatrix_0 FeatureMatrix #	
Trigger::=	FeatureMatrix FeatureMatrix #	
Trigger::=	{#,FeatureMatrix}	*end-of-string or a feature matrix*
Trigger::=	FeatureMatrix_0 {#,FeatureMatrix}	
Trigger::=	FeatureMatrix {#,FeatureMatrix}	
	Build feature matrices from constant phoneme or sequence of ±feature	
FeatureMatrix ::=	Phoneme	
FeatureMatrix ::=	[ ]	*empty feature matrix*
FeatureMatrix ::=	[+ Feature FeatureMatrix]	*+feature appended to matrix*
FeatureMatrix ::=	[- Feature FeatureMatrix]	*−feature appended to matrix*
Phoneme ::=	ə | a | g | ···	*a constant phoneme*
Feature ::=	voice | nasal | coronal | ···	*a phonological feature*
	integer indices are used for copying, see caption	
$\mathbb{Z}$ ::=	−2 | −1 | 1 | 2	*copying target, an integer*
α ::=	−2 | −1 | 1 | 2	*place copy target, an integer*

Figure S5: Context-free grammar generating phonological rules used by our system. Non-terminal symbols begin with a capital letter, as well as $\mathbb{Z}$ and α. For increased tractability, we arbitrarily bound the size of each FeatureMatrix to have had most three features, and as outlined in the above grammar over SPE rules, each trigger may have at most two feature matrices, hence the maximum range of copying targets ($\mathbb{Z}/\alpha$) of ±2. Copying targets are expressed as integers indexing into the triggering environments. For example the rule V → V_i / V_i_CC is expressed using the above grammar as V → -1 / V_CC, while the rule V → V_i / C_C_0V_i would be expressed as V → 2 / C_C_0V.

Rule::=	FC → FC / Trigger_Trigger
Rule::=	[+vowel] → [-highTone] / _[-vowel]* [+highTone +vowel]
Rule::=	[-sonorant] → [-voice] / _Trigger
Rule::=	[+nasal] → αplace / Trigger_[-vowel]
Rule::=	[+vowel] → [+highTone] / [+highTone +vowel] [-vowel]$_0$_Trigger
Rule::=	[+vowel] → [-back -low] / [-back +vowel] FeatureMatrix$_0$_
Rule::=	[-vowel] → [+voice] / _[-sonorant +voice]
Rule::=	FC → [+continuant] / FeatureMatrix_[+vowel]
Rule::=	[-sonorant] → FeatureMatrix / _[-sonorant]
Rule::=	[+voice] → FC / FeatureMatrix [-vowel]_Trigger
Rule::=	[-voice] → [+voice] / [+nasal] [-vowel]$_0$_
Trigger::=	#
Trigger::=	FeatureMatrix #
Trigger::=	FeatureMatrix$_0$ FeatureMatrix #
Trigger::=	FeatureMatrix FeatureMatrix #
Trigger::=	{#,FeatureMatrix}
Trigger::=	FeatureMatrix$_0$ {#,FeatureMatrix}
Trigger::=	FeatureMatrix {#,FeatureMatrix}
Trigger::=	
Trigger::=	FeatureMatrix
Trigger::=	FeatureMatrix* FeatureMatrix
Trigger::=	FeatureMatrix FeatureMatrix
Trigger::=	[-vowel]* [+highTone +vowel]
FeatureMatrix::=	+
FeatureMatrix::=	Phoneme
FeatureMatrix::=	[-sonorant]
FeatureMatrix::=	[+vowel +highTone]
FeatureMatrix::=	[-vowel]
FeatureMatrix::=	[-voice]
FeatureMatrix::=	[+vowel]
FeatureMatrix::=	[-back +vowel]
FeatureMatrix::=	[-sonorant +voice]
FeatureMatrix::=	[+nasal]
FeatureMatrix::=	[+voice]
FeatureMatrix::=	[+continuant -high]
FC::=	$\varnothing$
FC::=	$\mathbb{Z}$
FC::=	αplace
FC::=	FeatureMatrix

Figure S6: Symbolic structure of learned grammar over phonological rules. This grammar is estimated from a training set of 30 textbook problems selected for representativeness and diversity, and captures typically occurring motifs and schemas across grammars from the world's languages. Nonterminal FC refers to either the Focus or Change of a rule.

S3 Supplemental Methods

S3.1 Input data format

With the exception of allophony problems, each textbook problem consists of a matrix of surface forms, where the columns correspond to different inflections, and the rows correspond to different stems. Matrix entries can be empty (unspecified), either due to missing data, or due to a particular inflection not applying to a particular stem (for example, there is no past tense form of a stem like "pineapple" because it is a noun and not a verb). Slightly overloading notation used in the main manuscript, we refer to this matrix as **X**, and can index the rows of **X** (lexemes) and the columns of **X** (inflections). For example, Figure S7A shows a paradigm matrix **X** for a basic problem from Russian. There are 2 inflections (nominative and genitive), corresponding to the columns of the matrix, and 4 stems, corresponding to the rows of the matrix.

Allophony problems consist of a set of surface forms along with a set of pairs of phonemes, known as 'allophones', which we treat as a substitution on phonemes. Figure S7B shows an allophony problem from Mohawk.

A

	Nominative	Genitive
wagon	vagon	vagona
car	avtomobilʲ	avtomobilʲa
evening	vetʃer	vetʃera
husband	muʃ	muʒa
pencil	karandaʃ	karandaʃa
eye	glas	glaza
threshold	porok	poroga

B (i)

	Surface form
pigeon	oliːdeʔ
hide it! (sg.)	zahset
stocking	gaːlis
tail	odahsa
five	wisk
two	degeni
Abraham	aplam

B (ii)

Allophones 1	Allophones 2
b	p
d	t
g	k

Figure S7: **A**. An example paradigm matrix from Russian, which is a common format for the input data **X**. Columns correspond to different inflections (nominative, genitive) while the rows correspond to different stems (wagon, car, evening, ...). For example, [vagona] means *wagon* in the genitive form. **B**. Example allophone problem (a subset of data from Mohawk). A set of surface forms are given (**i**) together with pairs of possible allophones (**ii**), and the challenge is to find rule(s) which can predict one set of allophones from the other. There are 3 allophone pairs in this example.

S3.2 Counter-Example Guided Inductive Synthesis

We adapted counter-example guided inductive synthesis (CEGIS: [2]) to our setting ('CEGIS' in Figure 5) by maintaining (1) a 'current' theory, (2) a 'covered' set of rows of **X** for which there exists a stem such that the the current theory is consistent with that row; and then repeatedly (3) searching for the next counterexample, i.e. a row of **X** which is inconsistent with the current theory, and then using the Sketch program synthesizer to (4) update the current theory to be the MAP estimate of the theory that explains both this counterexample and the covered examples, and then (5) adding this most recent example to the 'covered' set. Because Sketch requires a finite program space we must also bound the number of rules searched over during step (4). We allow Sketch to search over at most $K + 1$ rules, where K is the number of rules in the most recent current theory.

S3.3 Incremental synthesis of grammars

Our incremental approach to synthesizing phonological grammars combines and generalizes counterexample guided synthesis with test-driven synthesis [3]. Similar to our CEGIS algorithm, we maintain a 'current' theory as well as a set of 'covered' examples, and repeatedly search for a (non-covered) counterexample to the current theory. However, rather than resolve from scratch for a new theory accommodating both the counterexample and the covered examples, we search only over those theories close in *edit distance* to the most recent theory.

We define the edit distance, $d(\mathbf{T}_1, \mathbf{T}_2)$, between a pair of theories $\mathbf{T}_1$, $\mathbf{T}_2$ by counting the number of insertions, deletions, substitutions, and swaps that separate the sequences of rules for $\mathbf{T}_1$ and $\mathbf{T}_2$. Any modification to a rule counts as a complete substitution, so entire rules are resynthesized wholesale rather than, e.g., have individual feature matrices 'edited'. This coarse-grained notion of edit distance has the advantage that it can be easily encoded in a SAT solver, and we hypothesize it may be less prone to getting trapped in local optima because it encourages larger search moves.

For each counterexample, we progressively increment the maximum edit distance until Sketch discovers a satisfying solution. We furthermore 'minibatch' counterexamples, grouped by lexeme (row of $\mathbf{X}$) and ordered according to the textbook problem. We automatically set the number of lexemes in each batch differently depending on the number of inflections (columns of $\mathbf{X}$) such that the surface forms in a minibatch will be no more than nine (ie, with 3 inflections, each minibatch will comprise 3 lexemes; with 4 inflections, each minibatch will comprise 2 lexemes). We conjecture that larger batch size generally leads to better convergence, because this exposes the SAT solver to more data at once, which on balance should lead to less myopic search moves. Yet larger batch sizes increase compute requirements, both because the size of the SAT problem grows linearly with batch size and because the search radius may need to grow larger with increased batch size. Accordingly, for allophony alternation problems, we batch the entire problem at once, because these problems are much easier. Our selection of a minibatch size of 9 was motivated by informal pilot experiments suggesting that after around 9 new words the solver performance degraded severely; due to the high compute cost of running these simulations, we did not perform a systematic hyperparameter sweep, and the 'optimal' batch size may differ from the one used.[2]

As a concrete example, consider a data set of English verb inflections in infinitive and third-person plurals. Suppose the batch size is two. If the first paradigm row is [mit] and [mits] (the pronunciations of the words "meet" and "meets"), then these are the first two words that the system considers. Initially, $\mathbf{T}_0$ contains no rules. So, these words serve as a counterexample, because the third-person-singular morphology, namely that a suffix must be appended (and that in the lexicon this suffix is recorded as /s/) has not yet been inferred. Running Sketch on this example would update the grammar to contain the third-person-singular suffix /s/, and introduce no new phonological rules. Suppose that the next paradigm row is [it] and [its] (the pronunciations of the words "eat" and "eats"). The system will find that the current grammar, when supplemented with the stem /it/, explains this example, and so it does not serve as a counterexample, because the morphophonology inferred from the first batch is consistent with this example. Suppose that the next row of the paradigm is [nid] and [nidz] (pronunciations of the words "need" and "needs"). There is no stem which can explain this paradigm row, given the current affixes and rules. Therefore it is a counterexample, and in the next iteration the system will accommodate this counterexample by introducing a phonological rule about explains the alternation between /s/ and /z/.

Parallelism. A naive implementation of this approach would encode the edit-distance constraint directly into the Sketch system. A more efficient, parallelizable approach is to enumerate a finite set of *theory templates*, where each template corresponds to a family of edits to the original theory. A *theory template* is a list, where each list element is either a fixed rule or a new unknown rule that the Sketch solver will solve for. The theory templates compatible with current rules $\{r_k\}_{k=1}^K$ is

$$\begin{aligned}\textsc{Templates}(\{r_k\}_{k=1}^K, D) = \Big\{\{r''_{k'}\}_{k'=1}^{K'} \quad : \quad & \underbrace{\forall \{r'_{k'}\}_{k'=1}^{K'} \text{ where } d(\{r'_{k'}\}_{k'=1}^{K'}, \{r_k\}_{k=1}^K) = D}_{\text{for all edits w/ edit distance } D} \\ & \text{and } \forall k' : \underbrace{r''_{k'} = \texttt{??} \text{ if } r'_{k'} \notin \{r_k\}_{k=1}^K}_{\text{new rule, denoted } \texttt{??}} \\ & \text{and } \forall k' : \underbrace{r''_{k'} = r'_{k'} \text{ if } r'_{k'} \in \{r_k\}_{k=1}^K}_{\text{fixed rule}} \Big\}\end{aligned} \quad (1)$$

where we have followed the convention, from Sketch, of writing ?? for unknown parts of the program that need to be synthesized. With this subroutine in hand we can define a constraint, $C_{\textsc{Template}}$, upon theories,

[2]Sketch requires further hyperparameters relating to converting an infinite program space to a finite one, such as an upper bound on the number of loop iterations. We set such upper bounds automatically so that the system can handle the longest surface form in the data: the loop iteration bound is the length of the longest surface form plus two, where +2 comes from a +1 buffer and another +1 due to implementation details.

datasets, and templates:

$$C_{\text{TEMPLATE}}(\mathbf{T},\mathbf{X},\{r_k\}_{k=1}^K) = C(\mathbf{T},\mathbf{X}) \wedge \forall k : (r_k \neq \texttt{??}) \Longrightarrow (r_k = \text{the } k^{\text{th}} \text{ rule in } \mathbf{T}) \tag{2}$$

where C imposes the constraint that $\mathbf{T}$ explains $\mathbf{X}$ (Eq. 4 of main text). This constraint can be passed to Sketch. We can now rewrite the incremental synthesis update equations from Eq. 6-7 of the main text to

$$D_{t+1} = \min_D D, \text{ such that } \exists\{r_k\}_{k=1}^K \in \text{TEMPLATES}(\mathbf{T}_t, D) \text{ and } C_{\text{TEMPLATE}}(\mathbf{T},\mathbf{X}_{t+1},\{r_k\}_{k=1}^K) \tag{3}$$

$$\mathbf{T}_{t+1} = \arg\max_{\mathbf{T}} F(\mathbf{X}_{t+1},\mathbf{T}), \text{ such that } \exists\{r_k\}_{k=1}^K \in \text{TEMPLATES}(\mathbf{T}_t, D_{t+1}) \text{ and } C_{\text{TEMPLATE}}(\mathbf{T},\mathbf{X}_{t+1},\{r_k\}_{k=1}^K) \tag{4}$$

This refactoring of our algorithm exposes an opportunity for parallelism: Given a specific value of $\{r_k\}_{k=1}^K$ drawn from $\text{TEMPLATES}(\mathbf{T}_t, D)$ we can straightforwardly solve the above equations using Sketch, so we simply loop over each such template and allocate a parallel worker to it. An outer loop monotonically increases the search distance D, and a parallel inner loop checks if any D-distance template satisfies C_{TEMPLATE}. When we find a D with such templates we return the best satisfying $\mathbf{T}$ under $F(\mathbf{X},\cdot)$.

We used 40 CPUs in our experiments. Unfortunately, our method *does not* saturate the parallel compute resources of many-core machines until the number of rules grows large. Therefore, we observe very little speed up on easier problems. This is because the number of distinct templates is smaller when there are fewer rules. For example, editing a theory with a single rule r_1 yields the templates $\{r'_1 = \texttt{??}\}$ and $\{r'_1 = \texttt{??}, r'_2 = \texttt{??}\}$, and so we only have two parallel jobs (other templates, such as $\{r'_1 = r_1, r'_2 = \texttt{??}\}$, are subsumed by the latter template).

S3.4 Bayesian prior over grammars

As a baseline UG, we simply count the number of symbols present in the lexicon and rules; we heuristically penalize insertions, deletions, and constant phonemes by counting them as two symbols:

$$\begin{aligned}
P(\mathbf{L}) &\propto \prod_{\langle f,c,m\rangle\in\mathbf{L}} \exp\left(-\text{length}(f)\right), \; f \text{ a form in the lexicon}\\
P(\mathbf{T}) &\propto \prod_{r\in\mathbf{T}} \exp\left(-\text{cost}(r)\right)\\
\text{cost}(\text{focus} \to \text{change / left_right}) &= \text{cost}(\text{focus}) + \text{cost}(\text{change}) + \text{cost}(\text{left}) + \text{cost}(\text{right})\\
\text{cost}(\varnothing) &= 2\\
\text{cost}(k) &= 2,\; k \text{ a constant phoneme}\\
\text{cost}([\pm\text{feature}_1 \;\cdots \pm\text{feature}_n]) &= 1+n\\
\text{cost}(\#) &= 1\\
\text{cost}(\{\#, m\}) &= 1 + \text{cost}(\#) + \text{cost}(m)\\
\text{cost}(m_0) &= 1 + \text{cost}(m)\\
\text{cost}(uv) &= \text{cost}(u) + \text{cost}(u), \text{ when } u,v \text{ elements of a trigger}
\end{aligned}$$

As a concrete example, the system solves the Russian problem in Fig. S7A using one rule:

$$\underbrace{\left[\text{ -son }\right]}_{\text{cost=1+1}} \to \underbrace{\left[\text{ -voice }\right]}_{\text{cost=1+1}} \;/\; \_\; \underbrace{\#}_{\text{cost=1}} \qquad \textit{total cost 5}$$

Therefore the total theory cost is 5, and the theory prior is $P(\mathbf{T}) \propto \exp(-5)$. This is defined up to a constant proportionality. The normalizing constant is guaranteed to be well-defined because Sketch only considers finite program spaces, hence a finite number of possible rule sequences. Because we only care about the relative probabilities of candidate grammars, we can ignore the normalizing constant.

In solving Fig. S7A the system also constructs a lexicon, which participates in the prior probability calculation. This contains affixes for the nominative and generative:

$$\mathbf{L} \supset \{\langle\epsilon, \texttt{pfx}, \text{NOMINATIVE}\rangle, \langle\epsilon, \texttt{pfx}, \text{GENITIVE}\rangle, \langle\epsilon, \texttt{sfx}, \text{NOMINATIVE}\rangle, \langle\text{a}, \texttt{sfx}, \text{GENITIVE}\rangle\}$$

where ϵ is the empty string. The total length of all affixes is $0 + 0 + 0 + 1 = 1$, contributing a factor of e^{-1} to $P(\mathbf{L})$. The latent stems also contribute factors to $P(\mathbf{L})$, because they are members of $\mathbf{L}$:

$$\mathbf{L} \supset \{\langle \text{vagon}, \texttt{stem}, \text{WAGON}\rangle, \langle \text{avtomobil}^{\text{j}}, \texttt{stem}, \text{CAR}\rangle, \langle \text{vet}^{\int}\text{er}, \texttt{stem}, \text{EVENING}\rangle,$$
$$\langle \text{muʒ}, \texttt{stem}, \text{HUSBAND}\rangle, \langle \text{karandaʃ}, \texttt{stem}, \text{PENCIL}\rangle, \langle \text{glaz}, \texttt{stem}, \text{EYE}\rangle, \langle \text{porog}, \texttt{stem}, \text{THRESHOLD}\rangle\}$$

Above, the total length of the listed stems is 5+9+5+3+8+4+5=39, which contributes an additional factor of e^{-39} to $P(\mathbf{L})$. If the Russian problem comprised only the data in Fig. S7A, then the prior probability of lexicon would be $P(\mathbf{L}) \propto e^{-39}e^{-1}$, the prior probability of the theory would be $P(\mathbf{T}) \propto e^{-5}$, and the total probability of the grammar would be $P(\mathbf{L}, \mathbf{T}) \propto e^{-39}e^{-1}e^{-5}$.

S3.5 Ablation studies

We studied several ablations[3] of our system; see Fig. 5. We found that basic representational concerns matter most: one *needs* the right rule representation, which we think of as being part of universal grammar. We studied the effect of changing the feature system, as well as the effect of ablating two key computational mechanisms (having features at all, and having Kleene star).

- We first changed the feature system from so-called 'articulatory' features to 'phonetic' features. Typically introductory phonology courses start by introducing phonetic features (features of sounds). Later one typically learns that these features can be more concisely and more generically expressed in terms of features of the motor actions required to produce those sounds ('articulation'). For example, *fricative* is an phonetic feature which becomes deprecated by the articulatory feature of *continuant*. Concretely, we took Odden's text *Introducing Phonology* [4] and identified the features used in the first five chapters as 'phonetic' features. Features in Chapter 6 onward we identify as 'articulatory features'.

  'Articulatory' features, as indicated by this textbook boundary, include: anterior, aspirated, back, bilabial, continuant, coronal, delayedRelease, flap, glide, high, highTone, lateral, liquid, long, low, lowTone, nasal, palatal, palletized, retroflex, risingTone, rounded, sibilant, sonorant, syllabic, tense, trill, unreleased, voice, vowel.

  'Phonetic' features, as indicated by this textbook boundary, include: affricate, alveolar, alveopalatal, aspirated, back, bilabial, central, dental, flap, fricative, front, glide, high, highTone, labiodental, laryngeal, lateral, liquid, long, low, lowTone, middle, nasal, palatal, palletized, pharyngeal, retroflex, rounded, sonorant, stop, syllabic, tense, trill, unreleased, uvular, velar, voice, vowel.

- For a more drastic demonstration of the centrality of basic computational mechanisms, we further ablate *all* phonological features. The system can still express rules in terms of specific phonemes, but cannot generalize and analogize across phonemes. We also remove Kleene star, which means not allowing the system to express rules whose triggers abstract over the number of times a phoneme occurs. Recall that this is notated with a subscript $_0$, thus all of our example rules with this subscript are unexpressible by this ablation. In principle, this ablation can still learn rules whose behavior is identical to the correct rules, simply by memorizing every phoneme for which a rule applies (due to the ablation of features), and every sequence length for which a rule applies (due to the ablation of Kleene star). In practice, the system no longer has the inductive bias to learn such generalizations; and furthermore, search becomes harder because the programs become much longer due to the need to memorize many specific cases.

S4 Full set of problems and model outputs

Allophony problems are given as a set of surface forms along with a set of pairs of phonemes. The goal of the student (as well as the goal of the model) is to recover rule(s) which predicts which element of each pair is the underlying form. Model outputs for alternation problems are of the form:

[3] An *ablation* is a variant of a system with components removed entirely or changed to be less powerful. Ablations are studied to understand the importance of the ablated components.

Surface form	UR
a given surface form	*model's predicted underlying form*
...	

The surface form...	*Is underlyingly...*
a phoneme	*phoneme*

Followed by a sequence of rules output by the model.

Non-allophony problems are given as a matrix of surface forms, where the columns range over different inflections and the rows range over different stems. Missing data is notated with a dash (−). We show the model's predicted concatenative morphology in the first row of each such matrix. In the penultimate column of each matrix we show the predicted underlying stems, and in the final column of each matrix we show the ground truth annotations. After each such matrix we show the rules output by the model. For example,

stem	stem+i	$\hat{\text{UR}}$	UR
klup	klubi	klub	**klub**
trup	trupi	trup	**trup**
...		...	

illustrates a problem with two inflections, where the input to the model is the data colored purple, from which it synthesizes the morphology and stems in salmon, which should be compared with the ground truth annotation in **bold**.

Manual rule grading: We evaluate the system's predicted rules on 15 randomly sampled problems, which are flagged with the text 'This problem was manually graded'. Notes from the grading process are provided for these problems. These notes specify the phonological processes (i.e. rules) that are deemed as 'ground truth'; which rules from the system correspond to those ground-truth processes; and which rules from the system are not part of the ground-truth solution (i.e. spurious rules).

Ewe:

Surface form	UR
zrɔ̃	zrɔ̃
ñra	ñra
lɔ̃	rɔ̃
kpla	kpra
mlagoo	mragoo
ɡblaa	ɡbraa
lolo	roro
wlu	wru
βla	βra
srɔ̃	srɔ̃
lãkle	rãkre
hle	hre
vlɔ	vrɔ
atra	atra
dru	dru
fle	fre
ɡlamaa	ɡramaa
litsa	ritsa
dzre	dzre
ɣla	ɣra
xloloo	xroroo
tsro	tsro
ɸle	ɸre
blema	brema
dɔlele	dɔrere
ŋlɔ	ŋrɔ
jre	jre
adoɡlo	adoɡro
kplu	kpru
kpali	kpari
klalo	kraro
atrakpoe	atrakpoe

The surface form...	*Is underlyingly...*
l	r

Rules:

$$\left[\ +\text{liquid}\ \right] \rightarrow \left[\ +\text{lateral}\ \right] / \{\begin{bmatrix}\text{-palletized}\\ \text{-coronal}\end{bmatrix},\#\}\_$$

Ganda:

Surface form	UR
kola	kola
lwana	lwana
buulira	buulila
lja	lja
luula	luula
omugole	omugole
lumonde	lumonde
eddwaliro	eddwalilo
oluganda	oluganda
olulimi	olulimi
wulira	wulila
beera	beela
jjukira	jjukila
erjato	eljato
omuliro	omulilo
effirimbi	effilimbi
emmeeri	emmeeli
eraddu	eladdu
wawaabira	wawaabila
lagira	lagila
ebendera	ebendela
leerwe	leelwe
luula	luula
ssaffaali	ssaffaali

The surface form...	*Is underlyingly...*
r	l

Rules:
$[\ \ ] \rightarrow [\text{ -lateral }] \ / \ [\text{ -back }]\_$

Papago:

Surface form	UR
bíᶾim	bídim
táːpan	táːpan
hídoḍ	hídoḍ
tᶴɨ́kid	tɨ́kid
gátwid	gátwid
tᶴúku	túku
dáɡşp	dáɡşp
tóha	tóha
dᶾúːki̽	dúːki̽
hɨ́wgid	hɨ́wgid
tᶴíhaŋ	tíhaŋ
tóñi	tóñi
wíḍut	wíḍut
táːtaḍ	táːtaḍ
kíːtᶴud	kíːtud
dóːdom	dóːdom
táːtam	táːtam

The surface form...	*Is underlyingly...*
dᶾ	d
tᶴ	t

Rules:

[-nasal, +coronal, -retroflex] → [-anterior, +sibilant] / _[+high]

Proto–Bantu:

Surface form	UR
βale	βale
leme	leme
taβe	taβe
pala	pala
kondɛ	konlɛ
zɔŋgɔ	zɔŋɣɔ
βɛɣa	βɛɣa
βɛmbe	βɛmβe
limo	limo
kaŋga	kaŋɣa
ɣɔmbɛ	ɣɔmβɛ
lelɔ	lelɔ
kiɣa	kiɣa
ɣiɣɛ	ɣiɣɛ
kulu	kulu
oŋgo	oŋɣo
tɛndɛ	tɛnlɛ
zala	zala
zɔɣu	zɔɣu
βele	βele
lɛlu	lɛlu
eɣi	eɣi
kiŋgɔ	kiŋɣɔ
nto	nto

The surface form...	*Is underlyingly...*
b	β
d	l
g	ɣ

Rules:

[ ] → d / n_

[ ] → [-continuant] / [+nasal]_[ ]

Mohawk:

Surface form	UR
oliːdeʔ	oliːteʔ
zahset	zahset
gaːlis	kaːlis
odahsa	otahsa
wisk	wisk
degeni	tekeni
aplam	aplam
ojaːgala	ojaːkala
ohjotsah	ohjotsah
labahbet	lapahpet
sduːha	stuːha
dᶾiks	dᶾiks
desdaʔn̥	testaʔn̥
deːzeknw̥	teːzeknw̥

The surface form...	*Is underlyingly...*
b	p
d	t
g	k

Rules:
[-continuant] → [+voice] / _[+vowel]

Quechua (Cuzco dialect):

Surface form	UR
qori	quri
t^{ʃ}oχlu	t^{ʃ}uχlu
qomir	qumir
niŋri	niŋri
moqo	muqu
hoqara	huqara
p^{h}ulju	p^{h}ulju
jujaŋ	jujaŋ
tulju	tulju
api	api
suti	suti
oɴqoj	uŋquj
t^{ʃ}ilwi	t^{ʃ}ilwi
t^{ʃh}it^{ʃ}iŋ	t^{ʃh}it^{ʃ}iŋ
t^{ʃh}aɴqaj	t^{ʃh}aŋqaj
aɴqosaj	aŋqusaj
qet^{ʃ}uŋ	qit^{ʃ}uŋ
pisqo	pisqu
musoχ	musuχ
t^{ʃ}uŋka	t^{ʃ}uŋka
jaɴqaŋ	jaŋqaŋ
t^{ʃ}ulju	t^{ʃ}ulju
qhelja	qhilja
qeɴqo	qiŋqu
t^{ʃ}eqaŋ	t^{ʃ}iqaŋ
qaŋ	qaŋ
noqa	nuqa
t^{ʃ}axra	t^{ʃ}axra
t^{ʃ}eχniŋ	t^{ʃ}iχniŋ
soχta	suχta
aχna	aχna
ljixlja	ljixlja
qosa	qusa
qara	qara
alqo	alqu
seɴqa	siŋqa
karu	karu
atoχ	atuχ
qaŋkuna	qaŋkuna
pusaχ	pusaχ
teχwaj	tiχwaj
t^{ʃ}aki	t^{ʃ}aki
wateχ	watiχ
aŋka	aŋka
waχtaj	waχtaj
haku	haku
waqaj	waqaj
kaŋka	kaŋka
waxt^{ʃ}a	waxt^{ʃ}a
waleχ	waliχ
t^{h}akaj	t^{h}akaj
reχsisqa	riχsisqa

The surface form...	*Is underlyingly...*
e	i
o	u
ɴ	ŋ

Rules:

$\left[\text{-continuant}\right] \rightarrow \textsc{n} \;/\; \_q$

$u \rightarrow o \;/\; \_\begin{bmatrix}\text{+back} \\ \text{-high}\end{bmatrix}$

$u \rightarrow o \;/\; q\_$

$i \rightarrow e \;/\; \left[\text{-palletized}\right]\_\left[\text{-anterior}\right]_0 \begin{bmatrix}\text{-nasal} \\ \text{+back}\end{bmatrix}$

Lhasa Tibetan:

Surface form	UR
aŋɡu	aŋku
aŋṭãã	aŋṭãã
aŋba	aŋpa
apsoo	apsoo
amt^{ʃ}ɔɔ	amt^{ʃ}ɔɔ
tukṭüü	tukṭüü
amto	amto
ɨɣu	ɨɣu
ɨmt^{ʃ}i	ɨmt^{ʃ}i
uṭɨ	uṭɨ
uβɯ	uβɯ
ea	ea
embo	empo
ʊʊtsi	ʊʊtsi
qa	ka
qaa	kaa
qaŋba	kaŋpa
qamba	kampa
qam	kam
qamtoo	kamtoo
qaaβo	kaaβo
kɨkṭi	kɨkṭi
kɨβu	kɨβu
kɨɨŋɡuu	kɨɨŋkuu
kik	kik
kiṭuu	kiṭuu
kɯcuu	kɯcuu
kɯrii	kɯrii
kiiɣuu	kiiɣuu
ku	ku
kupcɨ	kupcɨ
kupcaa	kupcaa
kɛnca	kɛnca
kɛmbo	kɛmpo
keɣöö	keɣöö
kerβa	kerβa
qo	ko
qomba	kompa
qɔr	kɔr
qɔɔɔɔ	kɔɔɔɔ
t^{ʃh}ea	t^{ʃh}ea
t^{ʃh}uɣum	t^{ʃh}uɣum
topcaa	topcaa
t^{h}oõõ	t^{h}oõõ
ṭaaãã	ṭaaãã
ṭuɣɨ	ṭuɣɨ
ṭuɴɢo	ṭuŋɡo
nɛɴɢaa	nɛŋɡaa
paɴɢɔɔ	paŋɡɔɔ
pɛɛβãã	pɛɛβãã
simɢãã	simɡãã

The surface form...	*Is underlyingly...*
b	p
d	t
ɢ	ɡ
ḍ	ṭ
ɴ	ŋ
q	k

g	k

Rules:

$$\begin{bmatrix} \text{+high} \end{bmatrix} \rightarrow \begin{bmatrix} \text{-sonorant} \\ \text{-high} \end{bmatrix} / \_\begin{bmatrix} \text{-nasal} \\ \text{+high} \end{bmatrix}_0 \begin{bmatrix} \text{+back} \\ \text{-high} \end{bmatrix}$$

$$\begin{bmatrix} \text{-coronal} \end{bmatrix} \rightarrow \begin{bmatrix} \text{+voice} \end{bmatrix} / \begin{bmatrix} \text{-vowel} \end{bmatrix}\_$$

Kikurai:

Surface form	UR
aβaánto	aβaánto
aβamúra	aβamúra
amahíı́ndi	amahíı́nri
amakɛ́ɛ́ndɔ	amakɛ́ɛ́nrɔ
eβã̌	eβã̌
eeŋgwé	eeŋɣwé
eɣã̌	eɣã̌
ekeβwɛ́	ekeβwɛ́
hoorá	hoorá
iβiɣúrúβe	iβiɣúrúβe
iβirúúŋgúuri	iβirúúŋɣúuri
uɣusı́ri	uɣusı́ri
βáinu	βáinu
βorjó	βorjó
itʃiiŋgɛ́na	itʃiiŋɣɛ́na
itʃiiŋgúrúβe	itʃiiŋɣúrúβe
ɣaβã̌	ɣaβã̌
itʃiiŋgúta	itʃiiŋɣúta
βɛrɛká	βɛrɛká
iɣitúúmbe	iɣitúúmβe
ɣúúká	ɣúúká
remã̌	remã̌
rɛɛntá	rɛɛntá
oβoɣááká	oβoɣááká
oβotééndééru	oβotéénrééru
okoɣéémbá	okoɣéémβá
okoómbára	okoómβára
okoβára	okoβára
okoóndɔ́ɣa	okoónrɔ́ɣa
okorɔ́ɣa	okorɔ́ɣa
romã̌	romã̌
teɣetá	teɣetá
ukuúmbuurjá	ukuúmβuurjá
uruɣúta	uruɣúta

The surface form...	*Is underlyingly...*
b	β
d	r
g	ɣ

Rules:

[-sonorant] → [-continuant] / [-vowel]_

r → d / n_

Modern Greek:

Surface form	UR
kano	kano
kori	kori
xano	xano
xori	xori
x^jino	xino
k^jino	kino
krima	krima
xrima	xrima
xufta	xufta
kufeta	kufeta
kali	kali
xali	xali
x^jeli	xeli
k^jeri	keri
x^jeri	xeri
ox^ji	oxi

The surface form...	*Is underlyingly...*
x^j	x
k^j	k

Rules:

$$\begin{bmatrix} \text{+back} \end{bmatrix} \rightarrow \begin{bmatrix} \text{+palletized} \end{bmatrix} / \_\begin{bmatrix} \text{-back} \\ \text{+tense} \end{bmatrix}$$

Farsi:

Surface form	UR
ær̃teʃ	ær̃teʃ
far̃si	far̃si
qædr̃i	qædr̃i
r̃ah	r̃ah
r̃ast	r̃ast
r̃iʃ	r̃iʃ
ahar̥̃	ahar̥̃
axær̥̃	axær̥̃
hær̃towr̥̃	hær̃towr̥̃
ʃir̥̃	ʃir̥̃
ahaři	ahar̃i
bæřadær̥̃	bær̃adær̥̃
t^{ʃ}eřa	t^{ʃ}er̃a
dařid	dar̃id
biřæng	bir̃æng
ʃiřini	ʃir̃ini

The surface form...	*Is underlyingly...*
ř	r̃

Rules:
$\left[\ \text{+liquid}\ \right] \rightarrow$ ř / $\left[\ \text{+vowel}\ \right]$_$\left[\ \text{+vowel}\ \right]$

Osage:

Surface form	UR
dábrĩ	ðábrĩ
áðik^hãʒã	áðik^hãʒã
da$t^ʃ$pé	ða$t^ʃ$pé
$t^ʃ$ʔéðe	$t^ʃ$ʔéðe
dakʔé	ðakʔé
ðéze	ðéze
dálĩ	ðálĩ
ðĩ́e	ðĩ́e
daʃtú	ðaʃtú
ðĩ́ʃki	ðĩ́ʃki

The surface form...	*Is underlyingly...*
d	ð

Rules:
[ ] → [-continuant] / _[+low] [ ]

Amharic:

Surface form	UR
fərəs	fərəs
tənəsa	tənəsa
jɛlɨʃlɨʃ	jəlɨʃlɨʃ
majɛt	majət
gənzəb	gənzəb
ʃɛgna	ʃəgna
nəñ	nəñ
məwdəd	məwdəd
mənnəsat	mənnəsat
məmkər	məmkər
ʒɛle	ʒəle
jɛlləm	jəlləm
mət^{ʃ}	mət^{ʃ}
məstət	məstət
fəlləgə	fəlləgə
agəññɛ	agəññə
təmət^{ʃ}t^{ʃ}ɛ	təmət^{ʃ}t^{ʃ}ə
mokkərə	mokkərə
kaʒʒɛ	kaʒʒə
ʒɛmmərə	ʒəmmərə
lat^{ʃ}t^{ʃ}ɛ	lat^{ʃ}t^{ʃ}ə
aʃʃɛ	aʃʃə
bəkkələ	bəkkələ
ʃɛməggələ	ʃəməggələ

The surface form...	*Is underlyingly...*
ɛ	ə

Rules:

$$ə \rightarrow ɛ \;/\; \begin{bmatrix} \text{-anterior} \\ \text{-liquid} \\ \text{-back} \end{bmatrix} \_$$

Gen:

Surface form	UR
agble	agble
agoŋglo	agoŋglo
aŋɔli	aŋɔli
akplɔ	akplɔ
sabulɛ	sabulɛ
sra	sla
alɔ	alɔ
atitrwɛ	atitlwɛ
avlɔ	avlɔ
blafogbe	blafogbe
drɛ	dlɛ
edrɔ	edlɔ
exlɔ	exlɔ
exle	exle
hlɛ	hlɛ
ŋlɔ	ŋlɔ
$t^{ʃ}$rɔ̃	$t^{ʃ}$lɔ̃
ñrã	ñlã
klɔ	klɔ
tre	tle
vlu	vlu
lɔ	lɔ
mla	mla
pleplelu	pleplelu
wla	wla
zro	zlo
esrɔ	eslɔ
etro	etlo
eñrɔ̃	eñlɔ̃
$d^{ʒ}$ro	$d^{ʒ}$lo

The surface form...	*Is underlyingly...*
r	l

Rules:
[ ] → [-lateral] / [+coronal]_

Kishambaa:

Surface form	UR
tagi	tagi
kitabu	kitabu
paalika	paalika
ni	ni
ŋombe	ŋombe
matagi	matagi
dodoa	dodoa
goʃa	goʃa
babu	babu
ndimi	ndimi
ŋgoto	ŋgoto
mbeu	mbeu
n̥t^{h}umbii	nt^{h}umbii
ŋok^{h}uŋguni	ŋok^{h}uŋguni
m̥p^{h}eho	mp^{h}eho

The surface form...	*Is underlyingly...*
m̥	m
n̥	n

Rules:

$$[\quad] \rightarrow [\ \text{-voice}\ ] \ / \ \_ \begin{bmatrix} \text{+aspirated} \\ \text{-back} \end{bmatrix}$$

Thai:

Surface form	UR
bil	bil
müü	müü
rak˺	rak˺
baa	baa
loŋ	loŋ
brüü	brüü
haa	haa
plaa	p˺laa
dii	dii
tᶴaan	tᶴaan
tʰee	tʰee
tʰruumɛɛn	tʰruumɛɛn
kʰɛŋ	kʰɛŋ
panjaa	p˺anjaa
ləəj	ləəj
pʰjaa	pʰjaa
lüak˺	lüak˺
klaaŋ	k˺laaŋ
tᶴʰat˺	tᶴʰat˺
traa	t˺raa
riip˺	riip˺
ɔɔk˺	ɔɔk˺
pʰrɛɛ	pʰrɛɛ
kiə	k˺iə
kʰwaa	kʰwaa
kɛɛ	k˺ɛɛ
draj	draj
düŋ	düŋ
kan	k˺an
tᶴuək˺	tᶴuək˺
pʰleeŋ	pʰleeŋ
tᶴʰan	tᶴʰan
staaŋ	st˺aaŋ
rap˺	rap˺
jiisip˺	jiisip˺
pʰaa	pʰaa
kʰaa	kʰaa
dam	dam
raaj	raaj
tit˺	t˺it˺
sip˺	sip˺
pen	p˺en

The surface form...	*Is underlyingly...*
p	p˺
k	k˺
t	t˺

Rules:
[ ] → [-unreleased] / _[ ]

Palauan:

Surface form	UR
kəðə	kəðə
bəðuk	bəðuk
ðiak	ðiak
diak	θiak
maθtŋoθ	maθtŋoθ
ðeːl	ðeːl
deːl	θeːl
ðiosəʔ	ðiosəʔ
diosəʔ	θiosəʔ
ðik	ðik
dik	θik
kuθ	kuθ
ʔoðiŋəl	ʔoðiŋəl
koaθ	koaθ
eaŋəθ	eaŋəθ
ŋərarəðə	ŋərarəðə
baθ	baθ
ieðlʔəðip	ieðlʔəðip
kəðeb	kəðeb
məðəŋei	məðəŋei
uðouθ	uðouθ
olðak	olðak

The surface form...	*Is underlyingly...*
d	θ

Rules:
[+coronal] → d / #_

Russian (in metatheory training set):

at+stem	bʲiz+stem	u+stem	ÛR	UR
atrózɨ	bʲizrózɨ	urózɨ	rózɨ	rózɨ
atálɨ	bʲizálɨ	uálɨ	álɨ	álɨ
atkaróvɨ	bʲizkaróvɨ	ukaróvɨ	karóvɨ	karóvɨ
adbaradɨ́	bʲizbaradɨ́	ubaradɨ́	baradɨ́	baradɨ́
atsʲistrɨ́	bʲissʲistrɨ́	usʲistrɨ́	sʲisʲtrɨ́	sʲistrɨ́

Rules:

$$\begin{bmatrix}\text{-sonorant}\end{bmatrix} \rightarrow \begin{bmatrix}\text{-voice}\end{bmatrix} / \_\begin{bmatrix}\text{+anterior}\end{bmatrix}$$

$$\begin{bmatrix}\text{-vowel}\end{bmatrix} \rightarrow \begin{bmatrix}\text{+voice}\end{bmatrix} / \_\begin{bmatrix}\text{-sonorant} \\ \text{+voice}\end{bmatrix}$$

Indonesian (in metatheory training set):

stem	mə+stem+i	ÛR	UR
lempar	məlempar	lempar	lempar
rasa	mərasa	rasa	rasa
wakil	məwakili	wakil	wakil
jakin	məjakini	jakin	jakin
masak	məmasak	masak	masak
nikah	mənikah	nikah	nikah
ŋaco	məŋaco	ŋaco	ŋaco
ɲaɲi	məɲaɲi	ɲaɲi	ɲaɲi
hituŋ	məŋhituŋ	ghituŋ	hituŋ
gambar	məŋgambar	ggambar	gambar
kirim	məŋirim	kirim	kirim
dəŋar	məndəŋar	ddəŋar	dəkar
tulis	mənulis	tulis	tulis
bantu	məmbantu	bbantu	bantu
pukul	məmukul	pukul	pukul
d^{ʒ}ahit	mən̆d^{ʒ}ahit	d^{ʒ}d^{ʒ}ahit	d^{ʒ}ahit
t^{ʃ}atat	mən̆t^{ʃ}atat	d^{ʒ}t^{ʃ}atat	t^{ʃ}atat
ambil	məŋambil	gambil	ambil
isi	məŋisi	gisi	isi
undaŋ	məŋundaŋ	gundaŋ	undaŋ

Rules:

$\left[\ +\text{vowel}\ \right] \to \varnothing\ /\ ə \left[\ \text{-glide}\ \right]_0 \_\#$

$\begin{bmatrix} \text{-sonorant} \\ +\text{voice} \end{bmatrix} \to \varnothing\ /\ \#\_$

$\left[\ \text{-sonorant}\ \right] \to \left[\ +\text{nasal}\ \right]\ /\ ə\_$

Yokuts :

stem+it	stem+hin	stem+nit	ÛR	UR
xatit	xathin	xatnit	xat	xat
gopit	gophin	gopnit	gop	gop
gijit	gijhin	gijnit	gij	gij
mutut	muthun	mutnut	mut	mut
sa:pit	saphin	sapnit	sa:p	sa:p
go:bit	gobhin	gobnit	go:b	go:b
me:kit	mekhin	meknit	me:k	me:k
ʔo:tut	ʔothun	ʔotnut	uʔo:t	uʔo:t
panat	pana:hin	pana:nit	pa:na:	pana:
hojot	hojo:hin	hojo:nit	ho:jo:	hojo:
ʔilet	ʔile:hin	ʔile:nit	ʔile:	ʔile:
cujot	cujo:hun	cujo:nut	cujo:	cujo:
paxa:tit	paxathin	paxatnit	paxa:t	paxa:t
ʔopo:tit	ʔopothin	ʔopotnit	ʔopo:t	ʔopo:t
hibe:jit	hibejhin	hibejnit	hibe:j	hibe:j
sudo:kut	sudokhun	sudoknut	sudo:k	sudo:k

Rules:

$$\begin{bmatrix}\text{-back}\\ \text{+vowel}\end{bmatrix} \rightarrow \text{u} \;/\; \text{u}\,[\quad]_0\_$$

$$[\quad] \rightarrow [\;\text{-long}\;] \;/\; \_[\quad]\,[\;\text{-vowel}\;]$$

$$[\;\text{+vowel}\;] \rightarrow \varnothing \;/\; \{[\;\text{+vowel}\;],\#\}\_$$

Turkish (in metatheory training set):

stem	stem+un	stem+lar	stem+larɨn	ÛR	UR
ip	ipin	ipler	iplerin	ip	ip
kɨz	kɨzɨn	kɨzlar	kɨzlarɨn	kɨz	kɨz
jüz	jüzün	jüzler	jüzlerin	jüz	jüz
pul	pulun	pullar	pullarɨn	pul	pul
el	elin	eller	ellerin	el	el
t^{ʃ}an	t^{ʃ}anɨn	t^{ʃ}anlar	t^{ʃ}anlarɨn	t^{ʃ}an	t^{ʃ}an
köj	köjün	köjler	köjlerin	köj	köj
son	sonun	sonlar	sonlarɨn	son	son

Rules:

$$\begin{bmatrix}\text{+vowel}\end{bmatrix} \rightarrow \begin{bmatrix}\text{-back}\\ \text{-low}\end{bmatrix} / \begin{bmatrix}\text{-back}\\ \text{+vowel}\end{bmatrix}\begin{bmatrix}\ \end{bmatrix}_0 \_$$

$$\begin{bmatrix}\text{+voice}\end{bmatrix} \rightarrow \begin{bmatrix}\text{-rounded}\end{bmatrix} / \begin{bmatrix}\text{-rounded}\end{bmatrix}\begin{bmatrix}\text{-vowel}\end{bmatrix}\_$$

Turkish :

stem	stem+k	ÛR	UR
ip	ipi	ip	ip
bit	biti	bit	bit
sebep	sebebi	sebib	sebib
kanat	kanadɨ	kanɨd	kanɨd
ʃeref	ʃerefi	ʃerif	ʃerif
kɨtᶴ	kɨtᶴɨ	kɨtᶴ	kɨtᶴ
pilot	pilotu	pilout	pilot
demet	demeti	demit	demet
ʃarap	ʃarabɨ	ʃarɨb	ʃarab
ahmet	ahmedi	ahmeid	ahmed
pabutᶴ	pabudᶾu	pabudᶾ	pabudᶾ
gütᶴ	güdᶾü	güdᶾ	güdᶾ
sepet	sepeti	sepit	sepet
sanat	sanatɨ	sanɨt	sanɨt
kep	kepi	keip	kep
kurt	kurdu	kurud	kurd
satᶴ	satᶴɨ	saɨtᶴ	satᶴ
renk	rengi	renig	reng

Rules:

[-sonorant] → [-voice] / _#

[ ] → [ ]$_i$ / [ ]$_i$ [-vowel]_{[+anterior],#}

[+high] → ∅ / [+sonorant]_[-vowel]

Bukusu (in metatheory training set):

stem+a	βa+stem+a	ñ+stem+a	ÛR	UR
t^{ʃ}a	βat^{ʃ}a	ñd^{ʒ}a	t^ʃ	t^ʃ
t^{ʃ}exa	βat^{ʃ}exa	ñd^{ʒ}exa	t^{ʃ}ex	t^{ʃ}ex
t^{ʃ}ut^{ʃ}uuŋga	βat^{ʃ}ut^{ʃ}uuŋga	ñd^{ʒ}ut^{ʃ}uuŋga	t^{ʃ}ut^{ʃ}uuñg	t^{ʃ}ut^{ʃ}uuŋg
talaanda	βatalaanda	ndalaanda	talaand	talaand
teexa	βateexa	ndeexa	teex	teex
tiira	βatiira	ndiira	tiir	tiir
piima	βapiima	mbiima	piim	piim
pakala	βapakala	mbakala	pakal	pakal
ketulula	βaketulula	ŋgetulula	ketulul	ketulul
kona	βakona	ŋgona	kon	kon
kula	βakula	ŋgula	kul	kul
kwa	βakwa	ŋgwa	kw	kw

Rules:

$$\left[\text{-voice}\right] \rightarrow \left[\text{+voice}\right] / \left[\text{+nasal}\right]\left[\text{-vowel}\right]_0\_$$

$$\left[\text{+nasal}\right] \rightarrow \alpha\text{place} / \_\begin{bmatrix}\alpha\text{place}\\ \text{-vowel}\end{bmatrix}$$

English (in metatheory training set):

stem	stem+d	stem+z	stem+ɪŋ	ÛR	UR
ro	rod	roz	roɪŋ	ro	ro
ʃo	ʃod	ʃoz	ʃoɪŋ	ʃo	ʃo
cal	cald	calz	calɪŋ	cal	cal
trn	trnd	trnz	trnɪŋ	trn	trn
græb	græbd	græbz	græbɪŋ	græb	græb
sim	simd	simz	simɪŋ	sim	sim
liv	livd	livz	livɪŋ	liv	liv
mʊv	mʊvd	mʊvz	mʊvɪŋ	mʊv	mʊv
həg	həgd	həgz	həgɪŋ	həg	həg
lʊk	lʊkt	lʊks	lʊkɪŋ	lʊk	lʊk
æsk	æskt	æsks	æskɪŋ	æsk	æsk
wɛrk	wɛrkt	wɛrks	wɛrkɪŋ	wɛrk	wɛrk
kɪs	kɪst	kɪsəz	kɪsɪŋ	kɪs	kɪs
fɪʃ	fɪʃt	fɪʃəz	fɪʃɪŋ	fɪʃ	fɪʃ
kwɪz	kwɪzd	kwɪzəz	kwɪzɪŋ	kwɪz	kwɪz
bʌz	bʌzd	bʌzəz	bʌzɪŋ	bʌz	bʌz
wet	wetəd	wets	wetɪŋ	wet	wet
wed	wedəd	wedz	wedɪŋ	wed	wed
nid	nidəd	nidz	nidɪŋ	nid	nid
lɪft	lɪftəd	lɪfts	lɪftɪŋ	lɪft	lɪft

Rules:

∅ → ə / [+sibilant]_z

∅ → ə / [-continuant, -sonorant, +coronal]_d

[-vowel] → [-voice] / [-voice]_#

Lithuanian (in metatheory training set):

at+stem+ti	ap+stem+ti	ÛR	UR
ateiti	–	ei	ei
atimti	–	im	im
atneʃti	–	neʃ	neʃ
atleisti	–	leis	leis
atlikti	–	lik	lik
atkoːpti	–	koːp	koːp
atpraʃiːti	–	praʃiː	praʃiː
atkurti	–	kur	kur
–	apeiti	ei	ei
–	apieʃkoːti	ieʃkoː	ieʃkoː
–	apakti	ak	ak
–	apmoːkiːti	moːkiː	moːkiː
–	aptemdiːti	temdiː	temdiː
–	apʃaukti	ʃauk	ʃauk
adbekti	–	bek	bek
adgauti	–	gau	gau
adbukti	–	buk	buk
adgimti	–	gim	gim
–	abgauti	gau	gau
–	abʒ^{j}ureti	ʒ^{j}ure	ʒ^{j}ure
–	abʒelti	ʒel	ʒel
–	abdauʒiːti	dauʒiː	dauʒiː
–	abdraskiːti	draskiː	draskiː

Rules:

$$\left[\text{ -vowel }\right] \rightarrow \left[\text{ +voice }\right] \;/\; \_\left[\begin{array}{l}\text{-sonorant}\\ \text{+voice}\end{array}\right]$$

Armenian (in metatheory training set):

k+stem+am	$\hat{\text{U}}$R	UR
kert^{h}am	ert^{h}	ert^{h}
kasiem	asi	asi
kaniem	ani	ani
kakaniem	akani	akani
koxniem	oxni	oxni
kurriem	urri	urri
kətam	kt	t
kəkienam	əkien	kien
gəbəzzam	gbəzz	bəzz
gəlam	gl	l
gəzəram	gzər	zər
k^{h}ət^{h}uojniem	k^{h}t^{h}uejni	t^{h}uojni
k^{h}ət^{ʃh}ap^{h}iem	k^{h}t^{ʃh}ap^{h}i	t^{ʃh}ap^{h}i
g^{h}əb^{h}ieřiem	g^{h}b^{h}ib^{h}ři	b^{h}iaři
g^{h}əg^{h}uom	g^{h}g^{h}u	g^{h}u
g^{h}əd^{zh}ieviem	g^{h}d^{zh}iavi	d^{zh}iavi

Rules:

[+vowel] $\rightarrow$ o / u_

[-vowel] $\rightarrow$ [-vowel]$_{i}$ / #_[-vowel]$_{i}$

[-vowel] $\rightarrow$ ə / [-vowel]_[-vowel]

[ ] $\rightarrow$ e / i_

Axininca Campa :

stem	no+stem+ti	ÛR	UR
toniro	notoniroti	toniro	toniro
jaarato	nojaaratoti	jaarato	jaarato
kanari	nojanariti	kanari	kanari
kosiri	nojosiriti	kosiri	kosiri
pisiro	nowisiroti	pisiro	pisiro
porita	noworitati	porita	porita

Rules:
k → j / [ ]_

p → w / [ ]_

This problem was manually graded:

- [–cor] → [–cons,+cont,+voi] / V_V (or possibly [–cor,–son]). The data is pathological in that the only place where these C's aren't V_V is word-initial, and it's cheaper to write "non-initial". Implemented by rules 1 and 2.

No spurious rules.

Kikuyu :

ko+stem+a	ÛR	UR
ɣotɛŋɛra	tɛŋɛr	tɛŋɛr
ɣokuua	kuu	kuu
ɣokoora	koor	koor
koruɣa	ruɣ	ruɣ
kooria	ori	ori
komɛɲa	mɛɲ	mɛɲ
kohɔta	hɔt	hɔt
ɣot^{ʃ}ina	t^{ʃ}in	t^{ʃ}in
koɣeera	ɣeer	ɣeer
koina	in	in
ɣot^{ʃ}uuka	t^{ʃ}uuk	t^{ʃ}uuk
ɣokaja	kaj	kaj
koɣaja	ɣaj	ɣaj

Rules:

$$\left[\ \text{-vowel}\ \right] \rightarrow \text{ɣ} \ /\ \#\_\left[\quad\right] \begin{bmatrix} \text{-low} \\ \text{-voice} \end{bmatrix}$$

$$\left[\ \text{-vowel}\ \right] \rightarrow \left[\ \text{+voice}\ \right] \ /\ \_\begin{bmatrix} \text{-sonorant} \\ \text{+voice} \end{bmatrix}$$

This problem was manually graded:

- More complex version, expressed as 2 rules: k → [+voi] / _V[-voi,–cont] followed by [+dor,+voi,–son]→[+cont] (because the data has no g, only ɣ). Or, more simply (with narrower coverage): k → ɣ / _V[-voi,–cont]. This is implemented by the first model output rule. Model rule 1 "incorrectly" changes all C's to ɣ, because there's no other data. Can't really fault it for that.

Rule 2 is spurious.

Korean (in metatheory training set):

stem+ə	stem+ko	ÛR	UR
ipə	ipko	ip	ip
kupə	kupko	kup	kup
kapʰa	kapko	kapʰ	kapʰ
cipʰə	cipko	cipʰ	cipʰ
tata	tatko	tat	tat
putʰə	putko	putʰ	putʰ
məkə	məkko	mək	mək
tʃukə	tʃukko	tʃuk	tʃuk
ikə	ikko	ik	ik
taka	takko	tak	tak
kaka	kakko	kak	kak
səkə	səkko	sək	sək

Rules:

[-sonorant] → [-aspirated] / _[-sonorant]

[+voice] → $[\ +\text{low}\ ]_i$ / $[\ +\text{low}\ ]_i$ [-vowel]_

Yowlumne :

stem+hin	stem+ka	stem+al	stem+it	$\hat{\text{UR}}$	UR
xathin	xatka	xatal	xatit	xat	xat
dubhun	dubka	dubal	dubut	dib	deb
xilhin	xilka	xilal	xilit	xil	xil
koʔhin	koʔko	koʔol	koʔit	koʔ	koʔ
doshin	dosko	doːsol	doːsit	doːs	doːs
ṣaphin	ṣapka	ṣaːpal	ṣaːpit	ṣaːp	ṣaːp
lanhin	lanka	laːnal	laːnit	laːn	laːn
mekhin	mekka	meːkal	meːkit	meːk	meːk
wonhin	wonko	woːnol	woːnit	woːn	woːn
paxathin	paxatka	paxaːtal	paxaːtit	paxaːt	paxaːt
hiwethin	hiwetka	hiweːtal	hiweːtit	hiweːt	hiweːt
ʔopothin	ʔopotko	ʔopoːtol	ʔopoːtit	ʔopoːt	ʔopoːt
jawalhin	jawalka	jawaːlal	jawaːlit	jawaːl	jawaːl
paʔiṭhin	paʔiṭka	paʔṭal	paʔṭit	paʔṭ	paʔṭ/paʔiṭ
ʔilikhin	ʔilikka	ʔilkal	ʔilkit	ʔilk	ʔilik/ʔilk
logiwhin	logiwka	logwol	logwit	logw	logiw/logw
ʔugunhun	ʔugunka	ʔugnal	ʔugnut	ʔugn	ʔugun/ʔugn
lihimhin	lihimka	lihmal	lihmit	lihm	lihm/lihim
ʔajijhin	ʔajijka	ʔajjal	ʔajjit	ʔajj	ʔajj/ʔajij
tojixhin	tojixka	tojxol	tojxit	tojx	tojix/tojx
lukulhun	lukulka	luklal	luklut	lukl	lukl/lukul
soːnilhin	soːnilka	sonlol	sonlit	soːnl	soːnil/soːnl
ʔaːmilhin	ʔaːmilka	ʔamlal	ʔamlit	ʔaːml	ʔaːmil/ʔaːml
moːjinhin	moːjinka	mojnol	mojnit	moːjn	moːjn/moːjin
ṣaːlikhin	ṣaːlikka	ṣalkal	ṣalkit	ṣaːlk	ṣaːlk/ṣaːlik

Rules:

$$\varnothing \rightarrow \text{i} \;/\; \left[\text{ -vowel }\right]\_\left[\text{ -vowel }\right]\left[\text{ -vowel }\right]$$

$$\text{a} \rightarrow \text{o} \;/\; \begin{bmatrix}\text{+rounded}\\ \text{-high}\end{bmatrix}\left[\text{ -vowel }\right]_0\_$$

$$\begin{bmatrix}\text{-back}\\ \text{+vowel}\end{bmatrix} \rightarrow \text{u} \;/\; \text{u}\left[\quad\right]_0\_$$

$$\left[\quad\right] \rightarrow \text{u} \;/\; \begin{bmatrix}\text{-sonorant}\\ \text{+voice}\end{bmatrix}\left[\text{ -voice }\right]_0\_\left[\text{ -continuant }\right]$$

$$\left[\quad\right] \rightarrow \left[\text{ -long }\right] \;/\; \_\left[\text{ -vowel }\right]\left[\text{ -vowel }\right]$$

This problem was manually graded:

- vowel epenthesis: $\varnothing \rightarrow$ i / C_CC (must precede rounding harmony, must precede vowel shortening). Model implements this with rule 1
- rounding harmony: [α high] $\rightarrow$ [β round] / [α high, β round] C_0_ (must follow epenthesis) (high vowels match preceding high in rounding (dub-hin$\rightarrow$dubhun), non-high match preceding non-high in rounding (doːs-al$\rightarrow$doːsol). Model gets these with rules 2 and 3 (model: front becomes round, not falsified because data has no examples of uC_0e)
- vowel shortening: Vː $\rightarrow$ V / _CC. Model implements this with rule 5

Rule 4 is spurious.

Hungarian (in metatheory training set):

stem	stem+ban	stem+toːl	stem+nak	ÛR	UR
kalap	kalabban	kalaptoːl	kalapnak	kalap	kalap
kuːt	kuːdban	kuːttoːl	kuːtnak	kuːt	kuːt
ʒaːk	ʒaːgban	ʒaːktoːl	ʒaːknak	ʒaːk	ʒaːk
reːs	reːzben	reːstöːl	reːsnek	reːs	reːs
ʃroːf	ʃroːvban	ʃroːftoːl	ʃroːfnak	ʃroːf	ʃroːf
lakaːʃ	lakaːʒban	lakaːʃtoːl	lakaːʃnak	lakaːʃ	lakaːʃ
ketret^{s}	ketred^{z}ben	ketret^{s}töːl	ketret^{s}nek	ketret^{s}	ketrat^{s}
test	tezdben	testtöːl	testnek	tezt	test
rab	rabban	raptoːl	rabnak	rab	rab
kaːd	kaːdban	kaːttoːl	kaːdnak	kaːd	kaːd
meleg	melegben	melektöːl	melegnek	melag	melag
viːz	viːzben	viːstöːl	viːznek	viːz	viːz
varaːʒ	varaːʒban	varaːʃtoːl	varaːʒnak	varaːʒ	varaːʒ
aːg^{j}	aːg^{j}ban	aːk^{j}toːl	aːg^{j}nak	aːg^{j}	aːg^{j}
sem	semben	semtöːl	semnek	sem	sem
bün	bünben	büntöːl	bünnek	bün	bün
toroñ	toroñban	toroñtoːl	toroñnak	toroñ	toroñ
fal	falban	faltoːl	falnak	fal	fal
öːr	öːrben	öːrtöːl	öːrnek	öːr	öːr
saːj	saːjban	saːjtoːl	saːjnak	saːj	saːj

Rules:

$$\left[\ +\text{vowel}\ \right] \rightarrow \begin{bmatrix} \text{-back} \\ \text{-low} \end{bmatrix} / \begin{bmatrix} \text{-back} \\ +\text{vowel} \end{bmatrix} \left[\quad\right]_0 \_$$

$$\left[\ \text{-vowel}\ \right] \rightarrow \left[\ +\text{voice}\ \right] / \_ \begin{bmatrix} \text{-sonorant} \\ +\text{voice} \end{bmatrix}$$

$$\left[\ \text{-sonorant}\ \right] \rightarrow \left[\ \text{-voice}\ \right] / \_ \left[\ \text{-voice}\ \right]$$

Kikuria (in metatheory training set):

stem+a	stem+era	ÛR	UR
suraaŋga	suraaŋgera	suraaŋg	suraaŋg
taaŋgata	taaŋgatera	taaŋgat	taaŋgat
baamba	baambera	baamb	baamb
reenda	reendera	riend	reend
rema	remera	rem	rem
hoora	hoorera	hoor	hoor
roma	romera	rom	rom
sooka	sookera	sook	sook
tat͡ʃora	tat͡ʃorera	tat͡ʃor	tat͡ʃor
siika	seekera	siik	siik
tiga	tegera	tig	tig
ruga	rogera	rug	rug
suka	sokera	suk	suk
huuta	hootera	huut	huut
riiŋga	reeŋgera	riiŋg	riiŋg
siinda	seendera	siind	siind

Rules:

$[\text{ +vowel }] \rightarrow [\text{ -high }] \;/\; \_[\text{ -liquid }]_0 \text{ e}$

Farsi :

stem	stem+an	$\hat{\text{UR}}$	UR
zæn	zænan	zæn	zæn
læb	læban	læb	læb
hæsud	hæsudan	hæsud	hæsud
bæradær	bæradæran	bæradær	bæradær
bozorg	bozorgan	bozorg	bozorg
mæleke	mælekean	mæleke	mæleke
valede	valedean	valede	valede
kæbire	kæbirean	kæbire	kæbire
ahu	ahuan	ahu	ahu
hamele	hamelean	hamele	hamele
bat^{ʃ}t^{ʃ}e	bat^{ʃ}t^{ʃ}egan	bat^{ʃ}t^{ʃ}eg	bat^{ʃ}t^{ʃ}eg
setare	setaregan	setareg	setareg
bænde	bændegan	bændeg	bændeg
azade	azadegan	azadeg	azadeg
divane	divanegan	divaneg	divaneg

Rules:
$\left[\text{ -vowel }\right] \rightarrow \varnothing$ / e_#

This problem was manually graded:

- g-deletion: g → ∅ / V_# (or e_#). Model rule 1 implements this process.

No spurious rules.

Tibetan (in metatheory training set):

stem	bd^{ʒ}u+stem	stem+bd^{ʒ}u	$\hat{\text{U}}$R	UR
d^{ʒ}u	–	–	bd^{ʒ}u	bd^{ʒ}u
d^{ʒ}ig	d^{ʒ}ugd^{ʒ}ig	–	gd^{ʒ}ig	gd^{ʒ}ig
ʃi	d^{ʒ}ubʃi	ʃibd^{ʒ}u	bʃi	bʃi
gu	d^{ʒ}urgu	gubd^{ʒ}u	rgu	rgu
ŋa	d^{ʒ}uŋa	ŋabd^{ʒ}u	ŋa	ŋa

Rules:

$\left[\text{ -vowel }\right] \rightarrow \varnothing$ / #_$\left[\text{ -vowel }\right]$

Makonde (in metatheory training set):

stem+áŋga	stem+íle	stem+a	ÛR	UR
amáŋga	amíle	áma	ám	ám
taváŋga	tavíle	táva	táv	táv
akáŋga	akíle	áka	ák	ák
patáŋga	patíle	póta	pót	pót
tatáŋga	tatíle	tóta	tót	tót
dabáŋga	dabíle	dóba	dób	dób
aváŋga	avíle	óva	óv	óv
amáŋga	amíle	óma	óm	óm
tapáŋga	tapíle	tépa	tép	tép
patáŋga	patíle	péta	pét	pét
aváŋga	avíle	éva	év	év
babáŋga	babíle	béba	béb	béb
utáŋga	utíle	úta	út	út
lukáŋga	lukíle	lúka	lúk	lúk
lumáŋga	lumíle	lúma	lúm	lúm
uŋgáŋga	uŋgíle	úŋga	úŋg	úŋg
iváŋga	ivíle	íva	ív	ív
pitáŋga	pitíle	píta	pít	pít
imbáŋga	imbíle	ímba	ímb	ímb
limáŋga	limíle	líma	lím	lím

Rules:

$$\begin{bmatrix} \text{+continuant} \\ \text{-high} \end{bmatrix} \rightarrow \text{a} \ / \ \_\begin{bmatrix} \text{-vowel} \end{bmatrix} \begin{bmatrix} \text{+stress} \\ \text{+vowel} \end{bmatrix}$$

$$\begin{bmatrix} \text{+vowel} \end{bmatrix} \rightarrow \begin{bmatrix} \text{-stress} \end{bmatrix} \ / \ \_\begin{bmatrix} \text{-vowel} \end{bmatrix}_0 \begin{bmatrix} \text{+stress} \\ \text{+vowel} \end{bmatrix}$$

North Saami :

stem	stem+in	ÛR	UR
varit	varihin	varih	varih
oahpis	oahpisin	oahpis	oahpis
tʃoarvuʃ	tʃoarvuʃin	tʃoarvuʃ	tʃoarvuʃ
lottaaʃ	lottaadʒin	lottaadʒ	lottaadʒ
tʃuoivvat	tʃuoivvagin	tʃuoivvag	tʃuoivvag
ahhkut	ahhkubin	ahhkub	ahhkub
suohkat	suohkaðin	suohkað	suohkað
heedʒoʃ	heedʒodʒin	heedʒodʒ	heedʒodʒ
aadʒdʒut	aadʒdʒubin	aadʒdʒub	aadʒdʒub
bissobeahtset	bissobeahtsehin	bissobeahtseh	bissobeahtseh
tʃeahtsit	tʃeahtsibin	tʃeahtsib	tʃeahtsib
jaaʔmin	jaaʔmimin	jaaʔmim	jaaʔmim
tʃuoivat	tʃuoivagin	tʃuoivag	tʃuoivag
laageʃ	laagedʒin	laagedʒ	laagedʒ
gahpir	gahpirin	gahpir	gahpir
gaauhtsis	gaauhtsisin	gaauhtsis	gaauhtsis
aaslat	aaslagin	aaslag	aaslag
baðooʃgaattset	baðooʃgaattsebin	baðooʃgaattseb	baðooʃgaattseb
ahhkit	ahhkiðin	ahhkið	ahhkið
bahaanaalat	bahaanaalagin	bahaanaalag	bahaanaalag
beʃtor	beʃtorin	beʃtor	beʃtor
heevemeahhtun	heevemeahhtunin	heevemeahhtun	heevemeahhtun
beedʒot	beedʒohin	beedʒoh	beedʒoh
bissomeahtun	bissomeahtumin	bissomeahtum	bissomeahtum
laðas	laðasin	laðas	laðas
heaijusmielat	heaijusmielagin	heaijusmielag	heaijusmielag
heaŋkkan	heaŋkkanin	heaŋkkan	heaŋkkan
jaman	jamanin	jaman	jaman

Rules:

$$\begin{bmatrix} \text{-liquid} \\ \text{-nasal} \\ \text{-sibilant} \end{bmatrix} \rightarrow \text{t} \;/\; \_\#$$

$$\begin{bmatrix} \text{-vowel} \end{bmatrix} \rightarrow \begin{bmatrix} \text{+coronal} \end{bmatrix} \;/\; \_\#$$

$$\begin{bmatrix} \text{-sonorant} \\ \text{+voice} \end{bmatrix} \rightarrow \text{ʃ} \;/\; \_\#$$

This problem was manually graded:

- coda place neutralization: [-syl] → [+cor] / _#
- final devoicing: [–son] → [–voi] / _# (possibly also intervocalic voicing: [–son,–cont] → [+voi] / V_V; but perhaps redundant with final devoicing)
- coda manner neutralization: [–son,αstrid] → [αcont] / _#. i.e. stridents→ s,ʃ, non-stridents→t (could be two rules)

According to grader, the model's outputs simulate the above processes, modulo differences in the feature system and lack of variable binding on features. No spurious rules.

Kerewe (in metatheory training set):

ku+ stem +a	ku+ stem +ana	ku+ stem +ila	ku+ stem +ilana	kutú+ stem +a	kukí+ stem +a	kutú+ stem +ila	kukítú+ stem +ila	ÛR	UR
kubala	kubalana	kubalila	kubalilana	kutúbála	kukíbála	kutúbálila	kukítúbalila	bal	bal
kugaja	kugajana	kugajila	kugajilana	kutúgája	kukígája	kutúgájila	kukítúgajila	gaj	gaj
kugula	kugulana	kugulila	kugulilana	kutúgúla	kukígúla	kutúgúlila	kukítúgulila	gul	gul
kubála	kubálána	kubálíla	kubálílana	kutúbála	kukíbála	kutúbálila	kukítúbalila	bál	bál
kulúma	kulúmána	kulúmíla	kulúmílana	kutúlúma	kukílúma	kutúlúmila	kukítúlumila	lúm	lúm
kusúna	kusúnána	kusúníla	kusúnílana	kutúsúna	kukísúna	kutúsúnila	kukítúsunila	sún	sún
kulába	kulábána	kulábíla	kulábílana	kutúlába	kukílába	kutúlábila	kukítúlabila	láb	láb

Rules:

$$\left[\ \text{+voice}\ \right] \rightarrow \left[\ \text{-highTone}\ \right] / \left[\ \text{+highTone}\ \right] \left[\ \text{-vowel}\ \right]\_$$

$$\left[\ \text{+vowel}\ \right] \rightarrow \left[\ \text{+highTone}\ \right] / \begin{bmatrix} \text{+highTone} \\ \text{+vowel} \end{bmatrix} \left[\ \text{-vowel}\ \right]_0\_\left[\ \text{-vowel}\ \right]$$

Polish :

stem	stem+i	ÛR	UR
klup	klubi	klub	klub
trup	trupi	trup	trup
dom	domi	dom	dom
snop	snopi	snop	snop
ʒwup	ʒwobi	ʒwob	ʒwob
trut	trudi	trud	trud
dzvon	dzvoni	dzvon	dzvon
kot	koti	kot	kot
lut	lodi	lod	lod
grus	gruzi	gruz	gruz
nos	nosi	nos	nos
vus	vozi	voz	voz
wuk	wugi	wug	wug
wuk	wuki	wuk	wuk
sok	soki	sok	sok
ruk	rogi	rog	rog
bur	bori	bor	bor
vuw	vowi	vow	vow
sul	soli	sol	sol
buj	boji	boj	boj
ʃum	ʃumi	ʃum	ʃum
ʒur	ʒuri	ʒur	ʒur

Rules:

$\left[\text{ +vowel }\right] \rightarrow \text{u} \;/\; \_\left[\begin{array}{c}\text{-nasal}\\ \text{+voice}\end{array}\right]\#$

$\left[\text{ -sonorant }\right] \rightarrow \left[\text{ -voice }\right] \;/\; \_\#$

This problem was manually graded:

- o-raising: o → [+high] / _[+voi,–nas]# (ordered before final devoicing). Model implements this with rule 1.
- Final devoicing: [–son] → [–voi] / _# (ordered after o-raising). Model implements this with rule 2.

No spurious rules.

Ancient Greek (in metatheory training set):

stem+s	stem+os	stem+i	stem+si	ÛR	UR
hals	halos	hali	halsi	hal	hal
ojs	ojos	oji	ojsi	oj	oj
sus	suos	sui	susi	su	su
kloːps	kloːpos	kloːpi	kloːpsi	kloːp	kloːp
p^{h}leːps	p^{h}leːbos	p^{h}leːbi	p^{h}leːpsi	p^{h}leːb	p^{h}leːb
kateːlips	kateːlip^{h}os	kateːlip^{h}i	kateːlipsi	kateːlip^{h}	kateːlip^{h}
p^{h}ulaks	p^{h}ulakos	p^{h}ulaki	p^{h}ulaksi	p^{h}ulak	p^{h}ulak
ajks	ajgos	ajgi	ajksi	ajg	ajg
salpiŋks	salpiŋgos	salpiŋgi	salpiŋksi	salpiŋg	salpiŋg
onuks	onuk^{h}os	onuk^{h}i	onuksi	onuk^{h}	onuk^{h}
t^{h}eːs	t^{h}eːtos	t^{h}eːti	t^{h}eːsi	t^{h}eːt	t^{h}eːt
k^{h}aris	k^{h}aritos	k^{h}ariti	k^{h}arisi	k^{h}arit	k^{h}arit
elpis	elpidos	elpidi	elpisi	elpid	elpid
korus	korut^{h}os	korut^{h}i	korusi	korut^{h}	korut^{h}
riːs	riːnos	riːni	riːsi	riːn	riːn
delp^{h}iːs	delp^{h}iːnos	delp^{h}iːni	delp^{h}iːsi	delp^{h}iːn	delp^{h}iːn

Rules:

$$\begin{bmatrix}\text{+anterior}\\ \text{+coronal}\end{bmatrix} \rightarrow \varnothing \;/\; \_\begin{bmatrix}\text{-vowel}\end{bmatrix}$$

$$\begin{bmatrix}\text{-sonorant}\end{bmatrix} \rightarrow \begin{bmatrix}\text{-aspirated}\\ \text{-voice}\end{bmatrix} \;/\; \_\begin{bmatrix}\text{-sonorant}\end{bmatrix}$$

Catalan (in metatheory training set):

stem	stem+ə	ÛR	UR
əkelj	əkeljə	əkelj	əkelj
mal	malə	mal	mal
siβil	siβilə	siβil	siβil
əskerp	əskerpə	əskerp	əskerp
ʃop	ʃopə	ʃop	ʃop
sɛk	sɛkə	sɛk	sɛk
əspɛs	əspɛsə	əspɛs	əspɛs
gros	grosə	gros	gros
baʃ	baʃə	baʃ	baʃ
koʃ	koʃə	koʃ	koʃ
tot	totə	tot	tot
brut	brutə	brut	brut
pɔk	pɔkə	pɔk	pɔk
prəsis	prəsizə	prəsiz	prəsiz
frənses	frənsezə	frənsez	frənsez
gris	grizə	griz	griz
kəzat	kəzaðə	kəzad	kəzad
bwit	bwiðə	bwid	bwid
rɔt^{ʃ}	rɔʒə	rɔd^{ʒ}	rɔd^{ʒ}
bot^{ʃ}	boʒə	bod^{ʒ}	bod^{ʒ}
orp	orβə	orb	orb
ljark	ljarɣə	ljarg	ljarg
sek	seɣə	seg	seg
fəʃuk	fəʃuɣə	fəʃug	fəʃug
grok	groɣə	grog	grog
puruk	puruɣə	purug	purug
kandit	kandiðə	kandid	kandid
frɛt	frɛðə	frɛd	frɛd
səɣu	səɣurə	səɣur	səɣur
du	durə	dur	dur
səɣəðo	səɣəðorə	səgəðor	səɣəðor
kla	klarə	klar	klar
nu	nuə	nu	nu
kru	kruə	kru	kru
flɔñd^{ʒ}u	flɔñd^{ʒ}ə	flɔñd^{ʒ}u	flɔñd^{ʒ}u
dropu	dropə	dropu	dropu
əgzaktə	əgzaktə	əgzaktə	əgzaktə
əlβi	əlβinə	əlβin	əlβin
sa	sanə	san	san
pla	planə	plan	plan
bo	bonə	bon	bon
sərɛ	sərɛnə	sərɛn	sərɛn
suβlim	suβlimə	suβlim	suβlim
al	altə	alt	alt
fɔr	fɔrtə	fɔrt	fɔrt
kur	kurtə	kurt	kurt
sor	sorðə	sord	sord
bɛr	bɛrðə	bɛrd	bɛrd
san	santə	sant	sant
kəlɛn	kəlɛntə	kəlɛnt	kəlɛnt
prufun	prufundə	prufund	prufund
fəkun	fəkundə	fəkund	fəkund
dəsen	dəsentə	dəsent	dəsent
dulen	dulentə	dulent	dulent
əstuðian	əstuðiantə	əstuðiant	əstuðiant
blaŋ	blaŋkə	blaŋr	blaŋk

Rules:

$$\begin{bmatrix} \text{-sonorant} \end{bmatrix} \rightarrow \begin{bmatrix} \text{-voice} \end{bmatrix} \;/\; \_\#$$

$$\text{t} \rightarrow \tilde{\text{n}} \;/\; \begin{bmatrix} \text{-vowel} \end{bmatrix}\_\#$$

$$\begin{bmatrix} \text{-sonorant} \\ \text{+voice} \end{bmatrix} \rightarrow \begin{bmatrix} \text{+continuant} \end{bmatrix} \;/\; \begin{bmatrix} \text{-nasal} \end{bmatrix}\_\begin{bmatrix} \text{+vowel} \end{bmatrix}$$

$$\begin{bmatrix} \text{-lateral} \\ \text{+sonorant} \\ \text{+coronal} \end{bmatrix} \rightarrow \varnothing \;/\; \_\#$$

$$\begin{bmatrix} \text{+vowel} \end{bmatrix} \rightarrow \varnothing \;/\; \begin{bmatrix} \text{-sonorant} \end{bmatrix}\_\text{ə}$$

$$\begin{bmatrix} \text{+nasal} \end{bmatrix} \rightarrow \text{k} \;/\; \begin{bmatrix} \text{-vowel} \end{bmatrix}\_$$

Serbo–Croatian (in metatheory training set):

stem	stem+a	stem+o	stem+i	stem+em	stem+l	stem+la	stem+ló	ÛR	UR
kriʒan	kriʒana	kriʒano	kriʒani	–	–	–	–	kriʒan	kriʒan
suntʃan	suntʃana	suntʃano	suntʃani	–	–	–	–	suntʃan	suntʃan
svetʃan	svetʃana	svetʃano	svetʃani	–	–	–	–	svetʃan	svetʃan
bogat	bogata	bogato	bogati	–	–	–	–	bogat	bogat
rapav	rapava	rapavo	rapavi	–	–	–	–	rapav	rapav
mlád	mladá	mladó	mladí	–	–	–	–	mlád	mlád
túp	tupá	tupó	tupí	–	–	–	–	túp	túp
blág	blagá	blagó	blagí	–	–	–	–	blág	blág
grúb	grubá	grubó	grubí	–	–	–	–	grúb	grúb
béo	belá	beló	belí	–	–	–	–	bél	bél
veseo	vesela	veselo	veseli	–	–	–	–	vesel	vesel
debéo	debelá	debeló	debelí	–	–	–	–	débél	debél
mío	milá	miló	milí	–	–	–	–	míl	míl
zelén	zelená	zelenó	zelení	–	–	–	–	zélen	zelén
kradén	kradená	kradenó	kradení	–	–	–	–	kradén	kradén
dalék	daleká	dalekó	dalekí	–	–	–	–	dálek	dalék
visók	visoká	visokó	visokí	–	–	–	–	vísók	visók
dubók	duboká	dubokó	dubokí	–	–	–	–	dúbók	dubók
jásan	jasná	jasnó	jasní	–	–	–	–	jásn	jásn
vázan	vaʒná	vaʒnó	vaʒní	–	–	–	–	váʒn	váʒn
sítan	sitná	sitnó	sitní	–	–	–	–	sítn	sítn
ledan	ledna	ledno	ledni	–	–	–	–	ledn	ledn
tának	tanká	tankó	tankí	–	–	–	–	tánk	tánk
krátak	kratká	kratkó	kratkí	–	–	–	–	krátk	krátk
blízak	bliská	bliskó	bliskí	–	–	–	–	blízk	blízk
úzak	uská	uskó	uskí	–	–	–	–	úzk	úzk
dóbar	dobrá	dobró	dobrí	–	–	–	–	dóbr	dóbr
óʃtar	oʃtrá	oʃtró	oʃtrí	–	–	–	–	óʃtr	óʃtr
bodar	bodra	bodro	bodri	–	–	–	–	bodr	bodr
ustao	ustala	ustalo	ustali	–	–	–	–	ustal	ustal
múkao	muklá	mukló	muklí	–	–	–	–	múkl	múkl
óbao	oblá	obló	oblí	–	–	–	–	óbl	óbl
pódao	podlá	podló	podlí	–	–	–	–	pódl	pódl
–	–	–	–	tepém	tépao	teplá	tepló	tép	tép
–	–	–	–	skubém	skúbao	skublá	skubló	skúb	zkúb
–	–	–	–	tresém	trésao	treslá	tresló	trés	trés
–	–	–	–	vezém	vézao	vezlá	vezló	véz	véz

Rules:

$$[\ \text{+vowel}\ ] \rightarrow [\ \text{+stress}\ ] \ / \begin{bmatrix}\text{+stress}\\ \text{+vowel}\end{bmatrix} [\quad]_0 \_$$

$$[\ \text{+vowel}\ ] \rightarrow [\ \text{-stress}\ ] \ / \ \_[\ \text{-vowel}\ ]_0 \begin{bmatrix}\text{+stress}\\ \text{+vowel}\end{bmatrix}$$

$$\varnothing \rightarrow \text{a} \ / \ [\ \text{-vowel}\ ]\_[\ \text{-vowel}\ ]\ \#$$

$$[\ \text{-sonorant}\ ] \rightarrow [\ \text{-voice}\ ] \ / \ \_[\ \text{-sonorant}\ ]$$

$$\text{l} \rightarrow \text{o} \ / \ \_\#$$

Russian (in metatheory training set):

stem	stem+a	$\hat{\text{UR}}$	UR
vagon	vagona	vagon	vagon
avtomobil^j	avtomobil^{j}a	avtomobil^j	avtomobil^j
vet^{ʃ}er	vet^{ʃ}era	vet^{ʃ}er	vet^{ʃ}er
muʃ	muʒa	muʒ	muʒ
karandaʃ	karandaʃa	karandaʃ	karandaʃ
glas	glaza	glaz	glaz
golos	golosa	golos	golos
ras	raza	raz	raz
les	lesa	les	les
porok	poroga	porog	porog
vrak	vraga	vrag	vrag
urok	uroka	urok	urok
porok	poroka	porok	porok
t^{s}vet	t^{s}veta	t^{s}vet	t^{s}vet
prut	pruda	prud	prud
soldat	soldata	soldat	soldat
zavot	zavoda	zavod	zavod
xlep	xleba	xleb	xleb
grip	griba	grib	grib
trup	trupa	trup	trup

Rules:
$\left[\text{ -sonorant }\right] \rightarrow \left[\text{ -voice }\right] / \_\#$

Finnish :

stem	stem+a	ÛR	UR
aamu	aamua	aamu	aamu
hopea	hopeaa	hopea	hopea
katto	kattoa	katto	katto
kello	kelloa	kello	kello
kirja	kirjaa	kirja	kirja
külmæ	külmææ	külmæ	külmæ
koulu	koulua	koulu	koulu
lintu	lintua	lintu	lintu
hüllü	hüllüæ	hüllü	hüllü
kömpelö	kömpelöæ	kömpelö	kömpelö
nækö	nææköæ	nækö	nækö
joki	jokea	joke	joke
kivi	kiveæ	kive	kive
muuri	muuria	muuri	muuri
naapuri	naapuria	naæpuri	naæpuri
nimi	nimeæ	nime	nime
kaappi	kaappia	kaappi	kaappi
kaikki	kaikkea	kaikke	kaikke
kiirehti	kiirehtiæ	kiirehti	kiirehti
lehti	lehteæ	lehte	lehte
mæki	mækeæ	mæke	mæke
ovi	ovea	ove	ove
posti	postia	posti	posti
tukki	tukkia	tukki	tukki
æiti	æitiæ	æiti	æiti
englanti	englantia	englanti	englanti
jærvi	jærveæ	jærve	jærve
koski	koskea	koske	koske
reki	rekeæ	reke	reke
væki	vækeæ	væke	væke

Rules:

$$[\quad] \rightarrow \begin{bmatrix} \text{-back} \\ \text{+vowel} \end{bmatrix} / +\_$$

$$\text{æ} \rightarrow \text{a} / \begin{bmatrix} \text{+back} \\ \text{+tense} \end{bmatrix} [\quad]_0\_$$

$$\text{e} \rightarrow \text{i} / \_\#$$

Kerewe (in metatheory training set):

ku+stem+a	m+stem+a	a+stem+a	stem+a	ÛR	UR
kupaamba	mpaamba	apaamba	paamba	paamb	paamb
kupaaŋga	mpaaŋga	apaaŋga	paaŋga	paaŋg	paaŋg
kupima	mpima	apima	pima	pim	pim
kupuupa	mpuupa	apuupa	puupa	puup	puup
kupeket͡ʃa	mpeket͡ʃa	apeket͡ʃa	peket͡ʃa	peket͡ʃ	peket͡ʃ
kupiinda	mpiinda	apiinda	piinda	piind	piind
kuhiiga	mpiiga	ahiiga	hiiga	hiig	hiig
kuheeka	mpeeka	aheeka	heeka	heek	heek
kuhaaŋga	mpaaŋga	ahaaŋga	haaŋga	haaŋg	haaŋg
kuheeba	mpeeba	aheeba	heeba	heeb	heeb
kuhiima	mpiima	ahiima	hiima	hiim	hiim
kuhuuha	mpuuha	ahuuha	huuha	huuh	huuh

Rules:
$\left[\text{ -voice }\right] \rightarrow \text{p} \;/\; \left[\text{ -vowel }\right]\_$

English (in metatheory training set):

stem+s	ÛR	UR
kæps	kæp	kæp
kæts	kæt	kæt
kaks	kak	kak
pruwfs	pruwf	pruwf
kæbz	kæb	kæb
kædz	kæd	kæd
kagz	kag	kag
hʊvz	hʊv	hʊv
flijz	flij	flij
plæwz	plæw	plæw
pjr̩ez	pjr̩e	pjr̩e
klæmz	klæm	klæm
kænz	kæn	kæn
karz	kar	kar
gəlz	gəl	gəl
slæps	slæp	slæp
hɪts	hɪt	hɪt
powks	powk	powk
stæbz	stæb	stæb
hajdz	hajd	hajd
dɪgz	dɪg	dɪg
læfs	læf	læf
pɪθs	pɪθ	pɪθ
slæmz	slæm	slæm
kænz	kæn	kæn
hæŋz	hæŋ	hæŋ
θrajvz	θrajv	θrajv
bejðz	bejð	bejð
flajz	flaj	flaj

Rules:
[-vowel] → [+voice] / [+voice]_#

Jita (in metatheory training set):

oku+ stem +a	oku+ stem +ira	oku+ stem +ana	oku+ stem +irana	okúmú+ stem +a	okúmú+ stem +ira	okutʃí+ stem +a	okútʃí+ stem +ira	ÛR	UR
okuβuma	okuβumira	okuβumana	okuβumirana	okumuβúma	okumuβúmira	okutʃiβúma	okutʃiβúmira	βum	βum
okusiβa	okusiβira	okusiβana	okusiβirana	okumusíβa	okumusíβira	okutʃisíβa	okutʃisíβira	siβ	siβ
okulúma	okulumíra	okulumána	okulumírana	–	–	–	–	lúm	lúm
okukúβa	okukuβíra	okukuβána	okukuβírana	–	–	–	–	kúβ	kúβ

Rules:

$$\left[\ \text{+vowel}\ \right] \rightarrow \left[\ \text{+highTone}\ \right] / \begin{bmatrix} \text{+highTone} \\ \text{+vowel} \end{bmatrix} \left[\ \text{-vowel}\ \right]_0 \_ \left[\ \text{-vowel}\ \right]$$

$$\left[\ \text{+vowel}\ \right] \rightarrow \left[\ \text{-highTone}\ \right] / \_ \left[\ \text{-vowel}\ \right]_0 \begin{bmatrix} \text{+highTone} \\ \text{+vowel} \end{bmatrix}$$

This problem was manually graded:

- Move a high tone one syllable to the right. Rules 1 and 2 interact to effect this change and no other change.

No spurious rules.

Koasati (in metatheory training set):

stem	am+stem	ÛR	UR
apahtᶴá	amapahtᶴá	apahtᶴá	apahtᶴá
asiktᶴí	amasiktᶴí	asiktᶴí	asiktᶴí
ilkanó	amilkanó	ilkanó	ilkanó
ifá	amifá	ifá	ifá
aːpó	amaːpó	aːpó	aːpó
iskí	amiskí	iskí	iskí
patᶴokkőka	ampatᶴokkőka	patᶴokkőka	patᶴokkőka
towá	antowá	towá	towá
kastó	aŋkastó	kastó	kastó
bajáːna	ambajáːna	bajáːna	bajáːna
táːta	antáːta	táːta	táːta
tᶴofkoní	añtᶴofkoní	tᶴofkoní	tᶴofkoní
kitiɬká	aŋkitiɬká	kitiɬká	kitiɬká
toní	antoní	toní	toní

Rules:

$$\left[\ \text{+nasal}\ \right] \rightarrow \alpha\text{place} \ / \ \_\left[\begin{array}{c}\alpha\text{place}\\ \text{-vowel}\end{array}\right]$$

Korean (in metatheory training set):

stem+ə	stem+nɨnta	ÛR	UR
ana	annɨnta	an	an
kama	kamnɨnta	kam	kam
sinə	sinnɨnta	sin	sin
t˺atɨmə	t˺atɨmnɨnta	t˺atɨm	t˺atɨm
nəmə	nəmnɨnta	nəm	nəm
nama	namnɨnta	nam	nam
tᶴʰama	tᶴʰamnɨnta	tᶴʰam	tᶴʰam
ipə	imnɨnta	ip	ip
kupə	kumnɨnta	kup	kup
tᶴəpə	tᶴəmnɨnta	tᶴəp	tᶴəp
tata	tannɨnta	tat	tat
putʰə	punnɨnta	putʰ	putʰ
tᶴotᶴʰa	tᶴonnɨnta	tᶴotᶴʰ	tᶴotᶴʰ
məkə	məŋnɨnta	mək	mək
sək˺ə	səŋnɨnta	sək˺	sək˺
tak˺a	taŋnɨnta	tak˺	tak˺
tᶴukə	tᶴuŋnɨnta	tᶴuk	tᶴuk
ikə	iŋnɨnta	ik	ik

Rules:

[+voice] → [+low]$_i$ / [+low]$_i$ [-vowel]_#

[-voice] → [+nasal] / _[-vowel]

[ ] → a / tᶴʰ_

Samoan (in metatheory training set):

stem	stem+ia	ÛR	UR
olo	oloia	olo	olo
lafo	lafoia	lafo	lafo
aŋa	aŋaia	aŋa	aŋa
usu	usuia	usu	usu
tau	tauia	tau	tau
taui	tauia	taui	taui
saːʔili	saːʔilia	saːʔili	saːʔili
vaŋai	vaŋaia	vaŋai	vaŋai
paʔi	paʔia	paʔi	paʔi
naumati	naumatia	naumati	naumati
saːuni	saːunia	saːuni	saːuni
seŋi	seŋia	seŋi	seŋi
lele	lelea	lele	lele
suʔe	suʔea	suʔe	suʔe
taʔe	taʔea	taʔe	taʔe
tafe	tafea	tafe	tafe
taːupule	taːupulea	taːupule	taːupule
palepale	palepalea	palepale	palepale
tuː	tuːlia	tuːl	tuːl
tau	taulia	taul	taul
ʔalo	ʔalofia	ʔalof	ʔalof
oso	osofia	osof	osof
sao	saofia	saof	saof
asu	asuŋia	asuŋ	asuŋ
pole	poleŋia	poleŋ	poleŋ
ifo	ifoŋia	ifoŋ	ifoŋ
ula	ulaŋia	ulaŋ	ulaŋ
milo	milosia	milos	milos
valu	valusia	valus	valus
vela	velasia	velas	velas
api	apitia	apit	apit
eʔe	eʔetia	eʔet	eʔet
lavaː	lavaːtia	lavaːt	lavaːt
uː	uːtia	uːt	uːt
puni	punitia	punit	punit
siʔo	siʔomia	siʔom	siʔom
ŋalo	ŋalomia	ŋalom	ŋalom
sopo	sopoʔia	sopoʔ	sopoʔ
au	aulia	aul	aul
maːtau	maːtaulia	maːtaul	maːtaul
ili	ilifia	ilif	ilif
ulu	ulufia	uluf	uluf
taŋo	taŋofia	taŋof	taŋof
soa	soaŋia	soaŋ	soaŋ
fesili	fesiliŋia	fesiliŋ	fesiliŋ
ʔote	ʔoteŋia	ʔoteŋ	ʔoteŋ
tofu	tofuŋia	tofuŋ	tofuŋ
laʔa	laʔasia	laʔas	laʔas
taŋi	taŋisia	taŋis	taŋis
motu	motusia	motus	motus
mataʔu	mataʔutia	mataʔut	mataʔut
sau	sautia	saut	saut
oʔo	oʔotia	oʔot	oʔot
ufi	ufitia	ufit	ufit
tanu	tanumia	tanum	tanum
moʔo	moʔomia	moʔom	moʔom
tao	taomia	taom	taom
fana	fanaʔia	fanaʔ	fanaʔ

Rules:

$$\begin{bmatrix} \text{-back} \\ \text{+vowel} \end{bmatrix} \rightarrow \varnothing \; / \; \begin{bmatrix} \text{-back} \\ \text{+vowel} \end{bmatrix} \_$$

$$\begin{bmatrix} \text{-vowel} \end{bmatrix} \rightarrow \varnothing \; / \; \_\#$$

Palauan :

mó+stem	stem+áll	stem+l	ÛR	UR
mədáŋəb	dəŋəbáll	dəŋóbl	dáŋób	daŋob
mətéʔəb	təʔəbáll	təʔíbl	téʔíb	teʔib
məŋétəm	ŋətəmáll	ŋətóml	ŋétóm	ŋetom
mətábək	təbəkáll	təbákl	tábák	tabak
məʔárəm	ʔərəmáll	ʔəróml	ʔáróm	ʔarom
məsésəb	səsəbáll	səsóbl	sésób	sesob

Rules:

$$\left[\text{ +voice }\right] \rightarrow \text{ə} \;/\; \left[\quad\right]\left[\text{ -vowel }\right]\_\left[\text{ -vowel }\right]\#$$

$$\left[\text{ +vowel }\right] \rightarrow \text{ə} \;/\; \_\left[\text{ -vowel }\right]_0 \begin{bmatrix}\text{+stress}\\ \text{+vowel}\end{bmatrix}$$

Tunica (in metatheory training set):

stem	stem+ʔuhki	stem+ʔɔki	stem+hkʔaki	ÛR	UR
pó	póʔuhki	póʔɔki	póhkʔaki	pó	pó
pí	píʔuhki	píʔɛki	píhkʔaki	pí	pí
já	jáʔuhki	jáʔaki	jáhkʔaki	já	já
tʃú	tʃúʔuhki	tʃúʔɔki	tʃúhkʔaki	tʃú	tʃú
hára	hárʔuhki	hárʔaki	hárahkʔaki	hárá	hára
hípu	hípʔuhki	hípʔɔki	hípuhkʔaki	hípu	hípu
náʃi	náʃʔuhki	náʃʔɛki	náʃihkʔaki	náʃi	náʃi

Rules:

$\begin{bmatrix} \text{+voice} \end{bmatrix} \rightarrow \text{ɛ} \,/\, \begin{bmatrix} \text{-back} \end{bmatrix} \begin{bmatrix} \text{-vowel} \end{bmatrix}\_\begin{bmatrix} \text{-sonorant} \end{bmatrix}$

$\begin{bmatrix} \text{+continuant} \\ \text{-high} \end{bmatrix} \rightarrow \text{a} \,/\, \text{á} \begin{bmatrix} \text{-vowel} \end{bmatrix}\_$

$\begin{bmatrix} \text{+voice} \end{bmatrix} \rightarrow \varnothing \,/\, \begin{bmatrix} \quad \end{bmatrix} \begin{bmatrix} \text{-vowel} \end{bmatrix}\_\text{ʔ}$

This problem was manually graded:

- vowel harmony: same backness and lowness as VC_: [–high,–low] → [αlow,βbk] / [αlow,βbk]C_ (ordered before V deletion). Model rules 1 and 2 implement this process but restrict the context in a way that's allowed because the data contains a pathologically tiny of front vowels, so the rule that it posits happens to work
- vowel deletion: V → ∅ / V[ˆʔ]_CV. Model rule 3 applies only to Cʔ_CV is fine. Application to [+voice] rather than V is allowed by such limited data, so that's OK

No spurious rules.

German (in metatheory training set):

stem	stem+ə	stem+ən	stem+ər	ÛR	UR
tak	tagə	–	–	tag	tag
volk	volkə	–	–	volk	volk
pəriskop	pəriskopə	–	–	pəriskop	pəriskop
hof	höfə	–	–	–	hof
wək	wəgə	–	–	wəg	wəg
ros	rosə	–	–	ros	ros
raup	–	raubən	–	raub	raub
ləit	–	ləidən	–	ləid	ləid
lop	–	lobən	–	lob	lob
lant	–	landən	–	land	land
rat	–	ratən	–	rat	rat
grəis	grəizes	–	–	grəiz	grəiz
braf	–	–	bravər	brav	brav

Rules:

$[\text{-sonorant}] \rightarrow [\text{-voice}]$ / _#

$[\text{-vowel}] \rightarrow [\text{+voice}]$ / _$\begin{bmatrix}\text{-sonorant}\\ \text{+voice}\end{bmatrix}$

$[\text{+voice}] \rightarrow$ s / $[\text{+high}]\ [\text{-vowel}]$_#

$\varnothing \rightarrow$ e / $\begin{bmatrix}\text{-sonorant}\\ \text{+voice}\end{bmatrix}$_$[\text{-voice}]$

Zoque (in metatheory training set):

stem	ŋ+stem	ÛR	UR
pama	mbama	pama	pama
tatah	ndatah	tatah	tatah
kwarto	ŋgwarto	kwarto	kwarto
plato	mblato	plato	plato
trama	ndrama	trama	trama
disko	ndisko	disko	disko
gaju	ŋgaju	gaju	gaju
t͡ʃoʔngoja	ɲd͡ʒoʔngoja	t͡ʃoʔngoja	t͡ʃoʔnkoja/t͡ʃoʔngoja
tsima	ndzima	tsima	tsima
sʌk	sʌk	sʌk	sʌk
faha	faha	faha	faha
ʃapun	ʃapun	ʃapun	ʃapun

Rules:

$$\left[\ \text{+nasal}\ \right] \rightarrow \varnothing \ / \ \#\_\begin{bmatrix}\text{+continuant}\\ \text{-high}\end{bmatrix}$$

$$\left[\ \text{+nasal}\ \right] \rightarrow \alpha\text{place} \ / \ \#\_\begin{bmatrix}\alpha\text{place}\\ \text{-vowel}\end{bmatrix}$$

$$\left[\ \text{-voice}\ \right] \rightarrow \left[\ \text{+voice}\ \right] \ / \ \left[\ \text{+nasal}\ \right]\left[\ \text{-vowel}\ \right]_0\_$$

This problem was manually graded:

- nasal deletion: [+nas] → ∅ / _[+cont]. Model gets this with rule 1
- nasal place assimilation: [+nas] → [α place] / _[α place,–syll]. Model gets this with rule 2.
- postnasal voicing: [-son] → [+voi] / [+nas]_. Model gets this with rule 3 (looks funny to allow voicing ts→dz, which should have been transcribed as a single segment)

No spurious rules.

Lumasaaba (in metatheory training set):

iŋ+stem	xa+stem	ÛR	UR
iɲjele	xaçele	çele	çele
iŋgaːfu	xaxaːfu	xaːfu	xaːfu
imbeβa	xaβeβa	βeβa	βeβa
iŋgoxo	xakoxo	koxo	koxo
iŋgwe	xakwe	kwe	kwe
indali	xatali	tali	tali
imboko	xaβoko	βoko	βoko

Rules:

$$\begin{bmatrix} \text{-sonorant} \end{bmatrix} \rightarrow \begin{bmatrix} \text{-continuant} \\ \text{+voice} \end{bmatrix} / \begin{bmatrix} \text{-vowel} \end{bmatrix}\_$$

$$\begin{bmatrix} \text{+nasal} \end{bmatrix} \rightarrow \alpha\text{place} / \_\begin{bmatrix} \alpha\text{place} \\ \text{-vowel} \end{bmatrix}$$

This problem was also manually graded:

- Post-nasal hardening: [–son] → [–cont] / [+nas]_. Model implements this process in first rule
- Post-nasal voicing: [–son] → [+voice] / [+nas]_. Model implements this process in first rule (according to manual grader: "nothing wrong with combining" the first and second processes into a single rule)
- Nasal place agreement: [+nas] → [α place] / _[α place,–syll]. Model implements this process in second rule.

Model outputs no spurious rules.

Russian :

stem+ú	stem+l	stem+la	stem+lʲíú	ÛR	UR
vʲirnú	vʲirnúl	vʲirnúla	vʲirnúlʲi	vʲirnú	vʲirnú
vrú	vrál	vralá	vrálʲi	vra	vrá
stajú	stajál	stajála	stajálʲi	stajá	stajá
pʲikú	pʲók	pʲiklá	pʲiklʲí	pʲók	pʲók
vʲizú	vʲós	vʲizlá	vʲizlʲí	–	vʲóz
magú	mók	maglá	maglʲí	–	móg
móknu	mók	mókla	móklʲi	–	mókn

Rules:

$$\begin{bmatrix}\text{+voice}\end{bmatrix} \rightarrow \begin{bmatrix}\text{+stress}\\ \text{+vowel}\end{bmatrix} / \begin{bmatrix}\text{-stress}\end{bmatrix}\begin{bmatrix}\text{-vowel}\end{bmatrix}\_\begin{bmatrix}\text{-vowel}\end{bmatrix}_0 \{\begin{bmatrix}\text{-back}\\ \text{+vowel}\end{bmatrix},\#\}$$

$$\begin{bmatrix}\text{+vowel}\end{bmatrix} \rightarrow \text{i} / \{\begin{bmatrix}\text{+stress}\\ \text{+vowel}\end{bmatrix},\#\}\begin{bmatrix}\text{-vowel}\end{bmatrix}\_\begin{bmatrix}\text{-vowel}\end{bmatrix}_0 \begin{bmatrix}\text{+stress}\\ \text{+vowel}\end{bmatrix}$$

$$\begin{bmatrix}\quad\end{bmatrix} \rightarrow \varnothing / \begin{bmatrix}\text{+continuant}\\ \text{-high}\end{bmatrix}\begin{bmatrix}\text{-lateral}\\ \text{-low}\end{bmatrix}\_\{\begin{bmatrix}\text{+vowel}\end{bmatrix},\#\}$$

Japanese :

stem+ru	stem+anai	stem+itai	stem+tda	stem+joo	ÛR	UR
neru	nenai	netai	neta	nejoo	ne	ne
miru	minai	mitai	mita	mijoo	mi	mi
ʃinu	ʃinanai	ʃinitai	ʃinda	ʃinoo	ʃin	ʃin
jomu	jomanai	jomitai	jonda	jomoo	jom	jom
jobu	jobanai	jobitai	jonda	joboo	job	job
kat^{s}u	katanai	kat^{ʃ}itai	katta	katoo	kat^ʃ	kat^s
kasu	kasanai	kaʃitai	kaʃita	kasoo	–	kas
waku	wakanai	wakitai	waita	wakoo	–	wak
t^{s}ugu	t^{s}uganai	t^{s}ugitai	t^{s}uida	t^{s}ugoo	–	t^{s}ug
karu	karanai	karitai	katta	karoo	–	kar
kau	kawanai	kaitai	katta	kaoo	–	kaw

Rules:

$$\begin{bmatrix}\text{-anterior}\\ \text{-low}\end{bmatrix} \rightarrow t^{s} \;/\; \begin{bmatrix}\text{+vowel}\end{bmatrix}\_\begin{bmatrix}\text{-vowel}\end{bmatrix}$$

$$\begin{bmatrix}\text{+voice}\end{bmatrix} \rightarrow n \;/\; \begin{bmatrix}\text{+vowel}\end{bmatrix}\_\begin{bmatrix}\text{-continuant}\end{bmatrix}$$

$$\begin{bmatrix}\text{-vowel}\end{bmatrix} \rightarrow \varnothing \;/\; \begin{bmatrix}\text{+vowel}\end{bmatrix}\begin{bmatrix}\text{-vowel}\end{bmatrix}\_$$

$$\begin{bmatrix}\text{-sonorant}\end{bmatrix} \rightarrow t \;/\; \begin{bmatrix}\text{-rounded}\\ \text{-nasal}\end{bmatrix}\_\begin{bmatrix}\text{-high}\end{bmatrix}$$

Swahili :

u+stem	stem	ma+stem	ÛR	UR
ubale	m̩bale	–	bale	bale
udago	n̩dago	–	dago	dago
ugimbi	ŋ̩gimbi	–	gimbi	gimbi
ud^{ʒ}ia	ɲ̩d^{ʒ}ia	–	d^{ʒ}ia	d^{ʒ}ia
upad^{ʒ}a	p^{h}ad^{ʒ}a	mapad^{ʒ}a	p^{h}ad^{ʒ}a	p^{h}ad^{ʒ}a
upamba	p^{h}amba	–	p^{h}amba	p^{h}amba
utunzo	t^{h}unzo	matunzo	t^{h}ŋzo	t^{h}unzo
utunda	t^{h}unda	–	t^{h}ŋda	t^{h}unda
ukelele	k^{h}elele	makelele	k^{h}elele	k^{h}elele
ukumbi	k^{h}umbi	–	k^{h}mbi	k^{h}umbi
ut^{ʃ}oma	t^{ʃh}oma	mat^{ʃ}oma	t^{ʃh}oma	t^{ʃh}oma
ut^{ʃ}aŋgo	t^{ʃh}aŋgo	–	t^{ʃh}aŋgo	t^{ʃh}aŋgo
ufuasi	fuasi	mafuasi	fuasi	fuasi
ufuko	fuko	–	fk^{h}o	fuko
uvuʃo	vuʃo	mavuʃo	vʃo	vuʃo
uvumbi	vumbi	–	vŋbi	vumbi
usiku	siku	masiku	siku	siku
usira	sira	–	sira	sira
uʃono	ʃono	maʃono	ʃono	ʃono
uʃaŋga	ʃaŋga	–	ʃaŋga	ʃaŋga
uwiŋgu	m̩biŋgu	–	bwiŋgu	biŋgu
uwili	m̩bili	–	bwili	wili
ulimi	n̩dimi	–	dlimi	limi
urefu	n̩defu	–	drefu	refu
umio	mio	–	mmio	mio
wimbo	ɲimbo	–	ɲiŋbo	imbo
wembe	ɲembe	–	ɲembe	embe
wakati	ɲakati	–	–	akati
uʃi	ɲuʃi	–	–	uʃi
ʃoka	–	maʃoka	–	ʃoka
tunda	–	matunda	–	tunda
kaʃa	–	makaʃa	–	kaʃa

Rules:

$$\begin{bmatrix}\text{-back}\\\text{-sibilant}\\\text{-tense}\end{bmatrix} \rightarrow \varnothing \;/\; \#\,[\quad]\_\begin{bmatrix}\text{-back}\\\text{+sonorant}\end{bmatrix}$$

$$\varnothing \rightarrow \text{m̩} \;/\; \#\_\begin{bmatrix}\text{-sonorant}\\\text{+voice}\end{bmatrix}\begin{bmatrix}\text{+vowel}\end{bmatrix}$$

$$\begin{bmatrix}\text{+nasal}\end{bmatrix} \rightarrow \alpha\text{place} \;/\; \_\begin{bmatrix}\alpha\text{place}\\\text{-vowel}\end{bmatrix}$$

$$\varnothing \rightarrow \text{u} \;/\; \begin{bmatrix}\text{-sonorant}\end{bmatrix}\_\begin{bmatrix}\text{-vowel}\end{bmatrix}$$

$$\begin{bmatrix}\text{-vowel}\end{bmatrix} \rightarrow \begin{bmatrix}\text{-aspirated}\end{bmatrix} \;/\; [\quad]\_$$

$$\begin{bmatrix}\text{+vowel}\end{bmatrix} \rightarrow \text{w} \;/\; \#\_\begin{bmatrix}\text{+vowel}\end{bmatrix}$$

Russian (in metatheory training set):

stem+á	stem	stem+έ	stem+ɨ	ÛR	UR
luná	lún	lunʲέ	lúnɨ	lún	lun
dɨrá	dɨ́r	dɨrʲέ	dɨ́rɨ	dɨ́r	dɨr
travá	tráf	travʲέ	trávɨ	tráv	trav
pʲilá	pʲíl	pʲilʲέ	pʲílɨ	pʲíl	pʲíl/pil
valná	vóln	valnʲέ	vólnɨ	–	voln
galavá	galóf	galavʲέ	gólavɨ	–	gólov
ʒɨlʲizá	ʒɨlʲós	ʒɨlʲizʲέ	ʒélʲizɨ	–	ʒélʲoz
ʒɨná	ʒón	ʒɨnʲέ	ʒónɨ	–	ʒon
zmʲijá	zmʲéj	zmʲijέ	zmʲéji	–	zmʲéj
mʲέna	mʲέn	mʲénʲi	mʲέnɨ	–	mʲέn
pʲilʲiná	pʲilʲón	pʲilʲinʲέ	pʲilʲinɨ́	–	pʲilón
bʲisʲέda	bʲisʲέt	bʲisʲédʲi	bʲisʲέdɨ	–	bʲisέd
bʲidá	bʲέt	bʲidʲέ	bʲέdɨ	–	bʲɛd
pʲitá	pʲát	pʲitʲέ	pʲitɨ́	–	pʲat
stʲiná	stʲέn	stʲinʲέ	stʲέnɨ	–	stɛn/stʲɛn
rʲiká	rʲέk	rʲikʲέ	rʲékʲi	–	rʲɛk/rɛk
slugá	slúk	slugʲέ	slúgʲi	–	slug
blaxá	blóx	blaxʲέ	blóxʲi	–	blox

Rules:

$$\left[\text{ -sonorant }\right] \rightarrow \left[\text{ -voice }\right] / \_\#$$

$$\left[\text{ -vowel }\right] \rightarrow \left[\text{ +palatalized }\right] / \_\begin{bmatrix}\text{-back}\\ \text{+vowel}\end{bmatrix}$$

$$\left[\text{ +vowel }\right] \rightarrow \left[\text{ -stress }\right] / \_\left[\text{ -vowel }\right]_0 \begin{bmatrix}\text{+stress}\\ \text{+vowel}\end{bmatrix}$$

This problem was manually graded. The textbook problem statement specifies that the rules governing stress are not to be found by the student; the system makes little headway because it gets stuck trying to explain the stress patterns.

- There are some rules for stress, but they're complicated and not easy (or possible?) to state in this formalism. This can't be solved with this data, in this model. A couple pieces that come close: suffix stress: delete in the context of a stem stress. stress: initial if no other stress (but there are exceptions, because it isn't really right).
- palatalization: $C \rightarrow C^j$ / _[-back]. Model implements this with rule 2.
- final devoicing: [–son] → [–voi] / _#. Model implements this with rule 1.
- vowel reduction: stressless V → ɨ / ʒ_. Model misses this process.
- vowel reduction: stressless o→a. Model misses this process.
- vowel reduction: stressless ɛ→i. Model misses this process.

Third rule (which moves stresses) is spurious, and occurs because the model is trying to explain stress patterns when the problem states that stress patterns do not need to be explained.

Finnish :

stem+n	stem	stem+t	stem+lta	stem+kna	ÛR	UR
kanadan	kanada	kanadat	kanadalta	kanadana	kanada	kanada
kirjan	kirja	kirjat	kirjalta	kirjana	kirja	kirja
aamun	aamu	aamut	aamulta	aamuna	aamu	aamu
talon	talo	talot	talolta	talona	talo	talo
koiran	koira	koirat	koiralta	koirana	koira	koira
hüvæn	hüvæ	hüvæt	hüvæltæ	hüvænæ	hüvæ	hüvæ
kuvan	kuva	kuvat	kuvalta	kuvana	kuva	kuva
lain	laki	lait	lailta	lakina	laki	laki
nælæn	nælkæ	nælæt	nælæltæ	nælkænæ	nælkæ	nælkæ
jalan	jalka	jalat	jalalta	jalkana	jalka	jalka
leuan	leuka	leuat	leualta	leukana	leuka	leuka
paran	parka	parat	paralta	parkana	parka	parka
reiæn	reikæ	reiæt	reiæltæ	reikænæ	reikæ	reikæ
nahan	nahka	nahat	nahalta	nahkana	nahka	nahka
vihon	vihko	vihot	viholta	vihkona	vihko	vihko
laihan	laiha	laihat	laihalta	laihana	laiha	laiha
avun	apu	avut	avulta	apuna	apu	apu
halvan	halpa	halvat	halvalta	halpana	halpa	halpa
orvon	orpo	orvot	orvolta	orpona	orpo	orpo
leivæn	leipæ	leivæt	leivæltæ	leipænæ	leipæ	leipæ
pæivæn	pæivæ	pæivæt	pæivæltæ	pæivænæ	pæiva	pæiva
kilvan	kilpa	kilvat	kilvalta	kilpana	kilpa	kilpa
külvün	külpü	külvüt	külvültæ	külpünæ	külpü	külpü
tavan	tapa	tavat	tavalta	tapana	tapa	tapa
korvan	korva	korvat	korvalta	korvana	korva	korva
æidin	æiti	æidit	æidiltæ	æitinæ	æiti	æiti
kodin	koti	kodit	kodilta	kotina	koti	koti
muodon	muoto	muodot	muodolta	muotona	muoto	muoto
tædin	tæti	tædit	tædiltæ	tætinæ	tæti	tæti
kadun	katu	kadut	kadulta	katuna	katu	katu
maidon	maito	maidot	maidolta	maitona	maito	maito
pöüdæn	pöütæ	pöüdæt	pöüdæltæ	pöütænæ	pöüta	pöütæ
tehdün	tehtü	tehdüt	tehdültæ	tehtünæ	tehtü	tehtü
læmmön	læmpö	læmmöt	læmmöltæ	læmpönæ	læmpö	læmpö
laŋŋan	laŋka	laŋŋat	laŋŋalta	laŋkana	laŋka	laŋka
sæŋŋün	sæŋkü	sæŋŋüt	sæŋŋültæ	sæŋkünæ	sæŋkü	sæŋkü
hinnan	hinta	hinnat	hinnalta	hintana	hinta	hinta
linnun	lintu	linnut	linnulta	lintuna	lintu	lintu
opinnon	opinto	opinnot	opinnolta	opintona	–	opinto
rannan	ranta	rannat	rannalta	rantana	ranta	ranta
luonnon	luonto	luonnot	luonnolta	luontona	luonto	luonto
punnan	punta	punnat	punnalta	puntana	punta	punta
tunnin	tunti	tunnit	tunnilta	tuntina	tunti	tunti
kunnon	kunto	kunnot	kunnolta	kuntona	kunto	kunto
kannun	kannu	kannut	kannulta	kannuna	–	kannu
linnan	linna	linnat	linnalta	linnana	–	linna
tumman	tumma	tummat	tummalta	tummana	–	tumma
auriŋŋon	auriŋko	auriŋŋot	auriŋŋolta	auriŋkona	auriŋko	auriŋko
reŋŋin	reŋki	reŋŋit	reŋŋiltæ	reŋkinæ	–	reŋki
vaŋŋin	vaŋki	vaŋŋit	vaŋŋilta	vaŋkina	vaŋki	vaŋki
kellon	kello	kellot	kellolta	kellona	kello	kello
kellan	kelta	kellat	kellalta	keltana	–	kelta
sillan	silta	sillat	sillalta	siltana	–	silta
kullan	kulta	kullat	kullalta	kultana	–	kulta
virran	virta	virrat	virralta	virtana	–	virta
parran	parta	parrat	parralta	partana	–	parta

Rules:

$[\text{+vowel}] \rightarrow [\text{-back}]$ / ü $[\text{-vowel}]_0$_

p $\rightarrow$ v / $[\ \ ]$_$[\ \ ]\ [\text{+coronal}]$

t $\rightarrow [\text{+voice}]$ / $[\ \ ]$_$[\ \ ]\ [\text{+coronal}]$

$[\text{+vowel}] \rightarrow [\text{-back}]$ / æ $[\ \ ]_0$_

$[\text{-vowel}] \rightarrow [\text{+nasal}]$ / $[\text{+nasal}]$_$[\ \ ]\ [\text{-back}]$

k $\rightarrow \varnothing$ / $[\ \ ]$_$[\ \ ]\ [\text{-high}]$

Korean :

(note: the data is shown transposed because otherwise it does not fit on the PDF page)

stem+ man	pamman	summan	pamman	pamman	namman	namman	namman	pamman
stem+ maŋk^{h}ɨm	pammaŋk^{h}ɨm	summaŋk^{h}ɨm	pammaŋk^{h}ɨm	pammaŋk^{h}ɨm	nammaŋk^{h}ɨm	nammaŋk^{h}ɨm	nammaŋk^{h}ɨm	pammaŋk^{h}ɨm
stem+ narɨm	pamnarɨm	sumnarɨm	pamnarɨm	pannarɨm	nannarɨm	nannarɨm	nannarɨm	pannarɨm
stem+	pap	sup	pam	pat	nat	nat	nat	pan
stem+ tero	paptero	suptero	pamtero	pattero	nattero	nattero	nattero	pantero
stem+ kwa	papkwa	supkwa	pamkwa	pakkwa	nakkwa	nakkwa	nakkwa	paŋkwa
stem+ pota	pappota	suppota	pampota	pappota	nappota	nappota	nappota	pampota
stem+ kat^{ʃ}i	papkat^{ʃ}i	supkat^{ʃ}i	pamkat^{ʃ}i	pakkat^{ʃ}i	nakkat^{ʃ}i	nakkat^{ʃ}i	nakkat^{ʃ}i	paŋkat^{ʃ}i
stem+ i	papi	sup^{h}i	pami	pat^{ʃh}i	nasi	nat^{ʃ}i	nat^{ʃh}i	pani
stem+ ɨn	papɨn	sup^{h}ɨn	pamɨn	pat^{h}ɨn	nasɨn	nat^{ʃ}ɨn	nat^{ʃh}ɨn	panɨn
stem+ e	pape	sup^{h}e	pame	pat^{h}e	nase	nat^{ʃ}e	nat^{ʃh}e	pane
stem+ ita	papita	sup^{h}ita	pamita	pat^{ʃh}ita	nasita	nat^{ʃ}ita	nat^{ʃh}ita	panita
stem+ ɨro	papɨro	sup^{h}ɨro	pamɨro	pat^{h}ɨro	nasɨro	nat^{ʃ}ɨro	nat^{ʃh}ɨro	panɨro
ÛR	pap	sup	pam	–	–	–	–	–
UR	pap	sup^h	pam	pat^h	nas	nat^ʃ	nat^{ʃh}	pan

Rules:

$[\ \text{-voice}\ ] \rightarrow [\ \text{+nasal}\ ]\ /\ \_[\ \text{+nasal}\ ]$

$[\ \text{-vowel}\ ] \rightarrow p^h\ /\ u\_[\ \text{+vowel}\ ]$

Sakha (Yakut) :

stem	stem+lar	stem+lɨɨn	ÛR	UR
aɣa	aɣalar	aɣalɨɨn	aɣa	aɣa
paarta	paartalar	paartalɨɨn	paarta	paarta
tɨa	tɨalar	tɨalɨɨn	tɨa	tɨa
kinige	kinigeler	kinigeliin	kinige	kinige
ʃie	ʃieler	ʃieliin	ʃie	ʃie
ije	ijeler	ijeliin	ije	ije
kini	kiniler	kiniliin	kini	kini
bie	bieler	bieliin	bie	bie
oɣo	oɣolor	oɣoluun	oɣo	oɣo
χopto	χoptolor	χoptoluun	χopto	χopto
börö	börölör	börölüün	börö	börö
tɨal	tɨallar	tɨallɨɨn	tɨal	tɨal
ɨal	ɨallar	ɨallɨɨn	ɨal	ɨal
kuul	kuullar	kuulluun	kuul	kuul
at	attar	attɨɨn	at	at
balɨk	balɨktar	balɨktɨɨn	balɨk	balɨk
ɨskaap	ɨskaaptar	ɨskaaptɨɨn	ɨskaap	ɨskaap
oɣus	oɣustar	oɣustuun	oɣɨs	oɣɨs
kus	kustar	kustuun	kus	kus
tünnük	tünnükter	tünnüktüün	tünnɨk	tünnɨk
sep	septer	septiin	sep	sep
et	etter	ettiin	et	et
örüs	örüster	örüstüün	örus	örus
tiis	tiister	tiistiin	tiis	tiis
soroχ	soroχtor	soroχtuun	soroχ	soroχ
oχ	oχtor	oχtuun	oχ	oχ
oloppos	oloppostor	oloppostuun	olappos	olappos
ötöχ	ötöχtör	ötöχtüün	ötöχ	ötöχ
ubaj	ubajdar	ubajdɨɨn	ubaj	ubaj
saraj	sarajdar	sarajdɨɨn	saraj	saraj
tɨj	tɨjdar	tɨjdɨɨn	tɨj	tɨj
atɨɨr	atɨɨrdar	atɨɨrdɨɨn	atɨɨr	atɨɨr
ojuur	ojuurdar	ojuurduun	ojɨur	ojɨur
ütʃügej	ütʃügejder	ütʃügejdiin	ütʃugaj	ütʃugaj
eʃiij	eʃiijder	eʃiijdiin	eʃiij	eʃiij
tomtor	tomtordor	tomtorduun	tomtor	tomtor
moɣotoj	moɣotojdor	moɣotojduun	moɣotoj	moɣotoj
kötör	kötördör	kötördüün	köter	köter
bölköj	bölköjdör	bölköjdüün	bölköj	bölköj
χatɨŋ	χatɨŋnar	χatɨŋnɨɨn	χatɨŋ	χatɨŋ
aan	aannar	aannɨɨn	aan	aan
tiiŋ	tiiŋner	tiiŋniin	tiiŋ	tiiŋ
sordoŋ	sordoŋnor	sordoŋnuun	sordoŋ	sordoŋ
olom	olomnor	olomnuun	olom	olom
oron	oronnor	oronnuun	oron	oron
bödöŋ	bödöŋnör	bödöŋnüün	bödeŋ	bödeŋ

Rules:

$$l \rightarrow d \ / \begin{bmatrix} \text{-lateral} \\ \text{-tense} \end{bmatrix} \_$$

$$\begin{bmatrix} \text{-vowel} \end{bmatrix} \rightarrow \begin{bmatrix} \text{-voice} \end{bmatrix} / \begin{bmatrix} \text{-voice} \end{bmatrix} \_$$

$$\begin{bmatrix} \text{+vowel} \end{bmatrix} \rightarrow \begin{bmatrix} \text{+rounded} \end{bmatrix} / \begin{bmatrix} \text{+rounded} \end{bmatrix} \begin{bmatrix} \text{-low} \end{bmatrix}_0 \_$$

$$\begin{bmatrix} \text{+continuant} \\ \text{-high} \end{bmatrix} \rightarrow \begin{bmatrix} \text{-rounded} \end{bmatrix} / \ u \begin{bmatrix} \text{-vowel} \end{bmatrix}_0 \_$$

$$\begin{bmatrix} \text{+vowel} \end{bmatrix} \rightarrow \begin{bmatrix} \text{-back} \\ \text{-low} \end{bmatrix} / \begin{bmatrix} \text{-back} \\ \text{+vowel} \end{bmatrix} \begin{bmatrix} \quad \end{bmatrix}_0 \_$$

$$\begin{bmatrix} \text{-sonorant} \\ \text{+voice} \end{bmatrix} \rightarrow \begin{bmatrix} \text{+nasal} \end{bmatrix} / \begin{bmatrix} \text{+nasal} \end{bmatrix} \_$$

Sadzava Ukrainian :

stem	stem+a	stem+i	stem	stem	stem	stem	stem	ÛR	UR
plast	plasta	plasʲkʲi	–	–	–	–	–	plast	plast
skorux	skoruxa	skorusʲi	–	–	–	–	–	skorux	skorux
ɣʲrʲix	ɣʲrʲixa	ɣʲrʲisʲi	–	–	–	–	–	ɣʲrʲix	ɣrix
pastux	pastuxa	pastusʲi	–	–	–	–	–	pastux	pastux
mʲnʲux	mʲnʲuxa	mʲnʲusʲi	–	–	–	–	–	mʲnʲux	mnʲux
pluɣ	pluɣa	pluzʲi	–	–	–	–	–	–	pluɣ
sʲtʲiɣ	stoɣa	stozʲi	–	–	–	–	–	–	stoɣ
sak	saka	satsʲi	–	–	–	–	–	–	sak
bek	bəka	bətsʲi	–	–	–	–	–	–	bek
lest	ləsta	ləsʲkʲi	–	–	–	–	–	–	lest
lest	lesta	lesʲkʲi	–	–	–	–	–	lest	lest
pʲlʲit	plota	plokʲi	–	–	–	–	–	–	plot
sʲmʲrʲid	smroda	smrogʲi	–	–	–	–	–	–	smrod
fʲist	fosta	fosʲkʲi	–	–	–	–	–	–	fost
mʲist	mosta	mosʲkʲi	–	–	–	–	–	–	most
lʲid	lædu	lədu	–	–	–	–	–	–	lod
dʲrʲit	drota	drokʲi	–	–	–	–	–	–	drot
mʲid	mædu	mədu	–	–	–	–	–	–	mæd
vʲil	vola	volʲi	–	–	–	–	–	–	vol
vʲiz	voza	vozʲi	–	–	–	–	–	–	voz
ser	sera	serʲi	–	–	–	–	–	–	ser
sʲnʲip	snopa	snopʲi	–	–	–	–	–	–	snop
ɣreb	ɣrəba	ɣrəbʲi	–	–	–	–	–	–	ɣreb
læbʲid	læbəda	læbəgʲi	–	–	–	–	–	–	læbæd
bærʲiɣ	bærəɣa	bærəzʲi	–	–	–	–	–	–	bæræɣ
pərʲiɣ	pəroɣa	pərozʲi	–	–	–	–	–	–	pəroɣ
porʲiɣ	poroɣa	porozʲi	–	–	–	–	–	–	poroɣ
bolʲek	bolʲəka	bolʲətsʲi	–	–	–	–	–	–	bolʲek
vorʲiɣ	voroɣa	vorozʲi	–	–	–	–	–	–	voroɣ
konək	konəka	konətsʲi	–	–	–	–	–	–	konək
potʲik	potoka	pototsʲi	–	–	–	–	–	–	potok
tʲik	toka	totsʲi	–	–	–	–	–	–	tok
kʲil	kola	kolʲi	–	–	–	–	–	–	kol
–	–	–	kovalʲ	kovalʲe	kovale	–	–	–	kovalʲ
–	–	–	dᶾmʲilʲ	dᶾmʲilʲe	dᶾmʲile	–	–	–	dᶾmilʲ
–	–	–	kʲrʲilʲ	kʲrʲilʲe	kʲrʲile	–	–	–	krilʲ
–	–	–	utᶴetəlʲ	utᶴetəlʲə	utᶴetələ	–	–	–	utᶴetəlʲ
–	–	–	græbʲinʲ	græbənʲə	græbənə	–	–	–	græbænʲ
–	–	–	olənʲ	olənʲə	olənə	–	–	–	olənʲ
–	–	–	jatᶴʲmʲinʲ	jatᶴmænʲə	jatᶴmænə	–	–	–	jatᶴmænʲ
–	–	–	jasʲinʲ	jasənʲə	jasənə	–	–	–	jæsænʲ
–	–	–	zʲekʲ	zʲekʲə	zʲetə	–	–	–	zʲetʲ
–	–	–	–	–	–	masʲkʲ	mastə	–	mastʲ
–	–	–	–	–	–	sʲmʲirʲkʲ	smærtə	–	smærtʲ
–	–	–	–	–	–	vʲisʲkʲ	vʲistə	–	vistʲ
–	–	–	–	–	–	ragʲisʲkʲ	radostə	–	radostʲ
–	–	–	–	–	–	sʲilʲ	solə	–	solʲ
–	–	–	–	–	–	poʃʲisʲkʲ	poʃəstə	–	poʃestʲ
–	–	–	–	–	–	zamʲikʲ	zamətə	–	zamætʲ
–	–	–	–	–	–	skatərʲkʲ	skatərtə	–	skatərtʲ
–	–	–	–	–	–	kʲisʲkʲ	kostə	–	kostʲ

Rules:

$$\begin{bmatrix} \text{-voice} \end{bmatrix} \rightarrow \text{k}^\text{j} \; / \; \_ \begin{bmatrix} \text{-back} \\ \text{+vowel} \end{bmatrix}$$

$$\begin{bmatrix} \text{-voice} \end{bmatrix} \rightarrow \text{s}^\text{j} \; / \; \begin{bmatrix} \text{+vowel} \end{bmatrix} \_ \begin{bmatrix} \text{+high} \end{bmatrix}$$

Standard Ukrainian :

stem	stem+am	stem+ov^{j}i	stem+i	stem+ov^{j}i	stem+o	stem+a	stem+u	stem+i	stem	ÛR	UR
zub	zubam	zubov^{j}i	zub^{j}i	–	–	–	–	–	–	zub	zub
sv^{j}it	sv^{j}itam	sv^{j}itov^{j}i	sv^{j}it^{j}i	–	–	–	–	–	–	sv^{j}it	sv^{j}it
z^{j}at^j	z^{j}at^{j}am	z^{j}atev^{j}i	–	z^{j}atev^{j}i	–	–	–	–	–	z^{j}at^j	z^{j}at^j
koʃ^{j}il^j	koʃel^{j}am	koʃelev^{j}i	koʃel^{j}i	–	–	–	–	–	–	koʃ^{j}il^j	koʃ^{j}el^j
zlod^{j}ij	zlod^{j}ijam	zlod^{j}ijev^{j}i	–	zlod^{j}ijev^{j}i	–	–	–	–	–	zlodij	zlod^{j}ij/zlo[illegible]
m^{j}is^{j}ar$^{s\ j}$	m^{j}is^{j}ar$^{s\ j}$am	m^{j}is^{j}at^{s}ev^{j}i	m^{j}is^{j}ar$^{s\ j}$i	–	–	–	–	–	–	m^{j}is^{j}ar$^{s\ j}$	m^{j}is^{j}ar$^{s\ j}$
korovaj	korovajam	korovajev^{j}i	korovaji	–	–	–	–	–	–	korovaj	korovaj
kam^{j}in^j	kamen^{j}am	kamenev^{j}i	kamen^{j}i	–	–	–	–	–	–	kam^{j}in^j	kam^{j}en^j
m^{j}id^j	m^{j}id^{j}am	m^{j}idev^{j}i	m^{j}id^{j}i	–	–	–	–	–	–	mid^j	mid^j
xl^{j}iw	xl^{j}ivam	xl^{j}ivov^{j}i	xl^{j}iv^{j}i	–	–	–	–	–	–	xliv	xliv/xl^{j}iv
holub	holubam	holubov^{j}i	holub^{j}i	–	–	–	–	–	–	holub	holub
s^{j}in	s^{j}inam	s^{j}inov^{j}i	s^{j}in^{j}i	–	–	–	–	–	–	sin	sin/s^{j}in
leb^{j}id^j	lebed^{j}am	lebedev^{j}i	lebed^{j}i	–	–	–	–	–	–	l^{j}eb^{j}id^j	leb^{j}ed^j
sus^{j}id	sus^{j}idam	sus^{j}idov^{j}i	–	sus^{j}idov^{j}i	–	–	–	–	–	susid	sus^{j}id
t^{ʃ}olov^{j}ik	t^{ʃ}olov^{j}ikam	t^{ʃ}olov^{j}ikov^{j}i	–	t^{ʃ}olov^{j}ikov^{j}i	–	–	–	–	–	t^{ʃ}olov^{j}ik	t^{ʃ}olov^{j}ik
l^{j}id	ledam	ledov^{j}i	led^{j}i	–	–	–	–	–	–	l^{j}id	l^{j}ed
bil^j	bol^{j}am	bolev^{j}i	bol^{j}i	–	–	–	–	–	–	bol^j	bol^j
riw	rovam	rovov^{j}i	rov^{j}i	–	–	–	–	–	–	rov	rov
stiw	stolam	stolov^{j}i	stol^{j}i	–	–	–	–	–	–	stol	stol
d^{j}id	d^{j}idam	d^{j}idov^{j}i	–	d^{j}idov^{j}i	–	–	–	–	–	did	d^{j}id
l^{j}it	l^{j}otam	l^{j}otov^{j}i	l^{j}ot^{j}i	–	–	–	–	–	–	l^{j}ot	l^{j}ot
mist	mostam	mostov^{j}i	most^{j}i	–	–	–	–	–	–	most	most
vet^{ʃ}ir	vet^{ʃ}oram	vet^{ʃ}orov^{j}i	vet^{ʃ}or^{j}i	–	–	–	–	–	–	v^{j}et^{ʃ}or	vet^{ʃ}or
–	–	–	–	–	t^{j}ilo	t^{j}ila	t^{j}ilu	t^{j}il^{j}i	t^{j}iw	til	t^{j}il
–	–	–	–	–	koleso	kolesa	kolesu	koles^{j}i	kol^{j}is	kol^{j}id	kol^{j}es
–	–	–	–	–	ozero	ozera	ozeru	ozer^{j}i	oz^{j}ir	oz^{j}or	oz^{j}er
–	–	–	–	–	selo	sela	selu	sel^{j}i	s^{j}iw	–	s^{j}el
–	–	–	–	–	pole	pol^{j}a	pol^{j}u	pol^{j}i	pil^j	–	pol^j
–	–	–	–	–	slovo	slova	slovu	slov^{j}i	sliw	slov	slov
–	–	–	–	–	more	mor^{j}a	mor^{j}u	mor^{j}i	mir^j	–	mor^j

Rules:

$$[\quad] \rightarrow e \;/\; [\text{ +palatalized }]\_[\text{ +voice }]\;[\quad]$$

$$[\text{ -vowel }] \rightarrow [\text{ +palatalized }] \;/\; \_\begin{bmatrix}\text{-back}\\ \text{+vowel}\end{bmatrix}$$

$$[\text{ -glide }] \rightarrow [\text{ -palatalized }] \;/\; \_e$$

$$\begin{bmatrix}\text{+continuant}\\ \text{-palatalized}\end{bmatrix} \rightarrow w \;/\; [\text{ -sibilant }]\;[\text{ +vowel }]\_\#$$

$$o \rightarrow i \;/\; \_[\text{ -vowel }]_0\;[\text{ -vowel }]\;\#$$

$$\begin{bmatrix}\text{-sonorant}\\ \text{+coronal}\end{bmatrix} \rightarrow \begin{bmatrix}\text{+continuant}\\ \text{+sibilant}\\ \text{-voice}\end{bmatrix} \;/\; k\;[\quad]_0\_$$

This problem was manually graded:

- v → w / _# (or: coda). Model has a /w/ rule, it's just not quite right; half credit for rule 4.
- C palatalization: C → C^j / _i (ordered after mid vowel raising). Model gets this with rule 2.
- depalatalization: C^j → C / _e. Model gets this with rule 3.
- o fronting: o → [-back] / _CC$_0$# (ordered before mid vowel raising). Model does fronting and raising in one fell swoop (rule 5), missing the opportunity to let the intermediate e trigger depalatalization. Half credit.
- mid vowel raising: e → i / _final syllable (ordered after C palatalization). Don't see anything the model does that's doing this for /e/. The model gets /o/ to i compiled into one rule, but we need raising for /e/ too: l^{j}ed→l^{j}id.

Rules 1 and 6 are spurious; 2 half-credit counts as one extra spurious for a total of three spurious rules.

Somali (in metatheory training set):

stem	stem+ta	stem+o	stem+aj	stem+taj	stem+naj	ÛR	UR
daar	daarta	daaro	–	–	–	daar	daar
gees	geesta	geeso	–	–	–	gees	gees
laf	lafta	lafo	–	–	–	laf	laf
lug	lugta	luɣo	–	–	–	lug	lug
naag	naagta	naaɣo	–	–	–	naag	naag
tib	tibta	tiβo	–	–	–	tib	tib
sab	sabta	saβo	–	–	–	sab	sab
bad	bada	baðo	–	–	–	bad	bad
ʃid	ʃida	ʃiðo	–	–	–	ʃid	ʃid
feeḍ	feeḍa	feeṛo	–	–	–	feeḍ	feeḍ
ʕiir	ʕiirta	ʕiiro	–	–	–	ʕiir	ʕiir
ʔul	ʔuʃa	ʔulo	–	–	–	ʔul	ʔul
bil	biʃa	bilo	–	–	–	bil	bil
meel	meeʃa	meelo	–	–	–	meel	meel
kaliil	kaliiʃa	kaliilo	–	–	–	kaliil	kaliil
najl	najʃa	najlo	–	–	–	najl	najl
sun	sunta	sumo	–	–	–	sum	sum
laan	laanta	laamo	–	–	–	laam	laam
sin	sinta	simo	–	–	–	sim	sim
dan	danta	dano	–	–	–	dan	dan
daan	daanta	daano	–	–	–	daan	daan
saan	saanta	saano	–	–	–	saan	saan
nirig	nirigta	nirgo	–	–	–	nirlg	nirg
jirid	jirida	jirdo	–	–	–	jirld	jird
hoɣol	hoɣoʃa	hoglo	–	–	–	hogll	hogl
gaβaḍ	gaβaḍa	gabḍo	–	–	–	gablḍ	gabḍ
baɣal	baɣaʃa	baglo	–	–	–	bagll	bagl
waħar	waħarta	waħaro	–	–	–	waħar	waħar
irbad	irbada	irbaðo	–	–	–	irbad	irbad
kefed	kefeda	kefeðo	–	–	–	kefed	kefed
ʃilin	ʃilinta	ʃilino	–	–	–	ʃilin	ʃilin
bohol	bohoʃa	boholo	–	–	–	bohol	bohol
ʔaajad	ʔaajada	ʔaajaðo	–	–	–	ʔaajad	ʔaajad
gaʕan	gaʕanta	gaʕmo	–	–	–	gaʕlm	gaʕm
ʔinan	ʔinanta	ʔinano	–	–	–	ʔinan	ʔinan
–	–	–	suɣaj	sugtaj	sugnaj	sug	sug
–	–	–	kaβaj	kabtaj	kabnaj	kab	kab
–	–	–	siðaj	sidaj	sidnaj	sid	sid
–	–	–	dilaj	diʃaj	dillaj	–	dil
–	–	–	ganaj	gantaj	gannaj	gan	gan
–	–	–	tumaj	tuntaj	tunnaj	–	tum
–	–	–	argaj	aragtaj	aragnaj	–	arg
–	–	–	gudbaj	guðubtaj	guðubnaj	–	gudb
–	–	–	qoslaj	qosoʃaj	qosollaj	–	qosl
–	–	–	hadlaj	haðaʃaj	haðallaj	–	hadl

Rules:

$[\text{-vowel}] \to [\ \ ]_i \ / \ [\ \ ]_i\, [\text{-vowel}]\_[\text{-vowel}]\, \{[\text{-vowel}],\#\}$

$[\text{-sonorant}] \to [\text{+continuant}] \ / \ [\text{+vowel}]\_[\text{+vowel}]$

$$[\text{-voice}] \to \varnothing \ / \ \begin{bmatrix} \text{-continuant} \\ \text{-nasal} \\ \text{+coronal} \end{bmatrix}\_$$

$[\text{-voice}] \to ʃ \ / \ \text{l}\_$

$\text{l} \to \varnothing \ / \ \_[\text{-vowel}]$

$[\text{+nasal}] \to \text{n} \ / \ \_\{[\text{-vowel}],\#\}$

This problem was manually graded:

- t-deletion: [–son,–cont,+cor] → ∅ / [–son,–cont,+cor]_. Rule 3 implements this process.
- lt coalescence: Implement in one of two ways. First way (not what model did): [+lat] → [+strid,–ant] / _[–son,–cont] (lt→ʃt) and [–son,–cont,+cor] → ∅ / [+strid,–ant]_ (t deletes, ordered second). Second way (what model did): [–son,+cor] → [+strid,–ant] / [+lat]_ (lt→lʃ) and [+lat] → ∅ / _[+strid,–ant] (ʃ deletes, ordered second). Rules 4 and 5 implement this process.
- V epenthesis: ∅ → [α high, β low, γ bk] / [–syll, α high, β low, γ bk]C_C#, must occur before spirantization. Model misses this process (and instead introduces the spurious first rule)
- Intervocalic spirantization: [–son] → [+cont] / [+syl]_[+syl] (ordered after epenthesis). Second rule implements this process.
- lateralization: [+nas,+cor] → [+lat] / [+lat]_ (ln→ll). Model misses this process.
- Nasal coda place neutralization: [+nas] → [+cor] / _$ (before syllable boundary, or before {C,#}). SPE didn't have 'syllable boundary', so {C,#} is a common context. Rule 6 implements this process.

Rule 1 is spurious.

Latin (in metatheory training set):

stem+s	stem+is	stem+iː	ÛR	UR
arks	arkis	–	ark	ark
duks	dukis	–	duk	duk
daps	dapis	–	dap	dap
reːks	reːgis	–	reːg	reːg
falanks	falangis	–	falang	falang
filiks	filikis	–	filik	filik
lapis	lapidis	–	lapid	lapid
liːs	liːtis	–	liːt	liːt
fraws	frawdis	–	frawd	frawd
noks	noktis	–	nokt	nokt
frons	frontis	–	front	front
frons	frondis	–	frond	frond
inkuːs	inkuːdis	–	inkuːd	inkuːd
sors	sortis	–	sort	sort
fuːr	fuːris	–	fuːr	fuːr
murmur	murmuris	–	murmur	murmur
augur	auguris	–	augur	augur
arbor	arboris	–	arbor	arbor
pugil	pugilis	–	pugil	pugil
sal	salis	–	sal	sal
adeps	adipis	–	adep	adep
apeks	apikis	–	apek	apek
priːnkeps	priːnkipis	–	briːnkep	priːnkep
ekwes	ekwitis	–	ekwet	ekwet
miles	militis	–	milet	milet
noːmen	noːminis	–	noːmen	noːmen
karmen	karminis	–	karmen	karmen
luːmen	luːminis	–	luːmen	luːmen
wenter	wentris	–	wente	wentr
pater	patris	–	pate	patr
kadaːwer	kadaːweris	–	kadaːwer	kadaːwer
tuːber	tuːberis	–	tuːbee	tuːber
piper	piperis	–	piper	piper
karker	karkeris	–	karker	karker
dieːs	–	dieːiː	dieː	dieː
liːber	–	liːberiː	liːbea	liːber
miser	–	miseriː	miser	miser
ager	–	agriː	age	agr
sinister	–	sinistriː	siniste	sinistr
liber	–	libriː	libe	libr
as	assis	–	as	ass
os	ossis	–	os	oss
far	farris	–	far	farr
mel	mellis	–	–	mell
oːs	oːris	–	–	oːs
floːs	floːris	–	–	floːs
muːs	muːris	–	–	muːs
cruːs	cruːris	–	–	kruːs
kinis	kineris	–	–	kinis
pulvis	pulveris	–	–	pulvis

Rules:

$$\begin{bmatrix} \text{+continuant} \\ \text{-high} \end{bmatrix} \rightarrow \text{r} \ / \ \text{e}\_$$

$$\begin{bmatrix} \text{+voice} \end{bmatrix} \rightarrow \begin{bmatrix} \text{+high} \end{bmatrix} \ / \ \begin{bmatrix} \quad \end{bmatrix} \begin{bmatrix} \text{-vowel} \end{bmatrix}\_\begin{bmatrix} \text{-continuant} \end{bmatrix} \begin{bmatrix} \text{+vowel} \end{bmatrix}$$

$$\begin{bmatrix} \text{-vowel} \end{bmatrix} \rightarrow \varnothing \ / \ \begin{bmatrix} \text{+sonorant} \\ \text{+coronal} \end{bmatrix}\_\#$$

$$\begin{bmatrix} \text{-sonorant} \end{bmatrix} \rightarrow \begin{bmatrix} \text{-voice} \end{bmatrix} \ / \ \_\begin{bmatrix} \text{-vowel} \end{bmatrix}$$

$$\begin{bmatrix} \text{+coronal} \end{bmatrix} \rightarrow \varnothing \ / \ \_\begin{bmatrix} \text{-vowel} \end{bmatrix} \#$$

$$\text{e} \rightarrow \text{r} \ / \ \_\begin{bmatrix} \text{+vowel} \end{bmatrix}$$

$$\varnothing \rightarrow \begin{bmatrix} \text{+continuant} \\ \text{-high} \end{bmatrix}_{\text{i}} \ / \ \{\text{f},\#\} \begin{bmatrix} \text{+continuant} \\ \text{-high} \end{bmatrix}\_\begin{bmatrix} \text{+continuant} \\ \text{-high} \end{bmatrix}_{\text{i}} \begin{bmatrix} \text{-back} \\ \text{+vowel} \end{bmatrix}$$

Turkish :

stem	stem+sɨ	stem+ja	stem+dan	stem+lar	ÛR	UR
oda	odasɨ	odaja	odadan	odalar	oda	oda
dere	deresi	dereje	dereden	dereler	dere	dere
ütü	ütüsü	ütüje	ütüden	ütüler	ütü	ütü
balo	balosu	baloja	balodan	balolar	balo	balo
arɨ	arɨsɨ	arɨja	arɨdan	arɨlar	arɨ	arɨ
laː	laːsɨ	laːja	laːdan	laːlar	laː	laː
binaː	binaːsɨ	binaːja	binaːdan	binaːlar	binaː	binaː
imlaː	imlaːsɨ	imlaːja	imlaːdan	imlaːlar	imlaː	imlaː
beː	beːsi	beːje	beːden	beːler	beː	beː
kep	kepi	kepe	kepten	kepler	kep	kep
at	atɨ	ata	attan	atlar	at	at
ek	eki	eke	ekten	ekler	ek	ek
ok	oku	oka	oktan	oklar	ok	ok
gütʃ	güdʒü	güdʒe	gütʃten	gütʃler	gütʃ	güdʒ
ahmet	ahmedi	ahmede	ahmetten	ahmetler	ahmet	ahmed
kurt	kurdu	kurda	kurttan	kurtlar	kurt	kurd
türk	türkü	türke	türkten	türkler	türk	türk
gentʃ	gentʃi	gentʃe	gentʃten	gentʃler	geʃtʃ	gentʃ
halk	halkɨ	halka	halktan	halklar	halk	halk
üst	üstü	üste	üstten	üstler	üst	üst
sarp	sarpɨ	sarpa	sarptan	sarplar	sarp	sarp
harp	harbɨ	harba	harptan	harplar	harb	harb
alt	altɨ	alta	alttan	altlar	alt	alt
renk	rengi	renge	renkten	renkler	reʃg	reng
his	hissi	hisse	histen	hisler	–	hiss
hür	hürrü	hürre	hürden	hürler	–	hürr
mahal	mahallɨ	mahalla	mahaldan	mahallar	–	mahall
hak	hakkɨ	hakka	haktan	haklar	–	hakk
zam	zammɨ	zamma	zamdan	zamlar	–	zamm
af	affɨ	affa	aftan	aflar	–	aff
arap	arabɨ	araba	araptan	araplar	arab	arab
kojun	kojunu	kojuna	kojundan	kojunlar	kojuʃ	kojun
pilot	pilotu	pilota	pilottan	pilotlar	–	pilot
kitap	kitabɨ	kitaba	kitaptan	kitaplar	–	kitap
domuz	domuzu	domuza	domuzdan	domuzlar	–	domuz
davul	davulu	davula	davuldan	davullar	–	davul
bajɨr	bajɨrɨ	bajɨra	bajɨrdan	bajɨrlar	–	bajɨr
somun	somunu	somuna	somundan	somunlar	somuʃ	somun
fikir	fikri	fikre	fikirden	fikirler	–	fikr
isim	ismi	isme	isimden	isimler	–	ism
bojun	bojnu	bojna	bojundan	bojunlar	–	bojn
tʃevir	tʃevri	tʃevre	tʃevirden	tʃevirler	–	tʃevr
devir	devri	devre	devirden	devirler	–	devr
kojun	kojnu	kojna	kojundan	kojunlar	–	kojn
karɨn	karnɨ	karna	karɨndan	karɨnlar	–	kam
burun	burnu	burna	burundan	burunlar	–	burn
akɨl	aklɨ	akla	akɨldan	akɨllar	–	akl
ʃehir	ʃehri	ʃehre	ʃehirden	ʃehirler	–	ʃehr
namaz	namazɨ	namaza	namazdan	namazlar	–	namaz
zaman	zamaːnɨ	zamaːna	zamandan	zamanlar	–	zamaːn
harap	haraːbɨ	haraːba	haraptan	haraplar	–	haraːb
iːkaz	iːkaːzɨ	iːkaːza	iːkazdan	iːkazlar	–	iːkaːz
hajat	hajaːtɨ	hajaːta	hajattan	hajatlar	–	hajaːt
ispat	ispaːtɨ	ispaːta	ispattan	ispatlar	–	ispaːt
inek	inei	inee	inekten	inekler	–	inek
mantɨk	mantɨɨ	mantɨa	mantɨktan	mantɨklar	–	mantɨk
ajak	ajaɨ	ajaa	ajaktan	ajaklar	–	ajak
tʃabuk	tʃabuu	tʃabua	tʃabuktan	tʃabuklar	–	tʃabuk
dakik	dakii	dakie	dakikten	dakikler	–	dakik
merak	meraːkɨ	meraːka	meraktan	meraklar	–	meraːk
tebrik	tebriːki	tebriːke	tebrikten	tebrikler	–	tebriːk

hukuk	hukuːku	hukuːka	hukuktan	hukuklar	–	hukuːk

Rules:

$$[\text{-vowel}] \rightarrow \varnothing \;/\; [\text{-sonorant}]\_[\;\;]\#$$

$$\text{ɨ} \rightarrow \text{u} \;/\; [\text{+rounded}]\,[\text{-vowel}]_0\_$$

$$\begin{bmatrix}\text{-continuant}\\ \text{+coronal}\end{bmatrix} \rightarrow [\text{+voice}] \;/\; [\text{-low}]\,[\text{+voice}]\_$$

$$[\text{-sonorant}] \rightarrow [\text{-voice}] \;/\; \_\{[\text{-vowel}],\#\}$$

$$[\text{+vowel}] \rightarrow \begin{bmatrix}\text{-back}\\ \text{-low}\end{bmatrix} \;/\; \begin{bmatrix}\text{-back}\\ \text{+vowel}\end{bmatrix}[\text{-nasal}]_0\_$$

$$\text{ʃ} \rightarrow [\text{+nasal}]$$

$$[\text{-sonorant}] \rightarrow [\text{-voice}] \;/\; [\text{-voice}]\_$$

Kera :

stem+en	stem+em	stem+i	stem+u	stem+a	stem+eŋ	ÛR	UR
haman	hamam	hɨmi	hɨmu	hama	hamaŋ	hɨm	ham
seːnen	seːnem	siːni	siːnu	seːna	seːneŋ	siːn	seːn
kolon	kolom	kuli	kulu	kola	koloŋ	kul	kol
giːdin	giːdim	giːdi	giːdu	giːdɨ	giːdiŋ	giːd	giːd
cɨːrɨn	cɨːrɨm	ciːri	cuːru	cɨːrɨ	cɨːrɨŋ	–	cɨːr
gunun	gunum	guni	gunu	gunɨ	gunuŋ	–	gun
bɨlan	bɨlam	bɨli	bɨlu	bɨla	bɨlaŋ	–	bal
ŋɨfan	ŋɨfam	ŋɨfi	ŋɨfu	ŋɨfa	ŋɨfaŋ	–	ŋaf
ʔasan	ʔasam	ʔɨsi	ʔɨsu	ʔasa	ʔasaŋ	–	ʔas
ʔapan	ʔapam	ʔɨpi	ʔɨpu	ʔapa	ʔapaŋ	–	ʔap
haran	haram	hɨri	hɨru	hara	haraŋ	–	har
balnan	balnam	bɨlni	bɨlnu	balna	balnaŋ	–	baln
ŋafnan	ŋafnam	ŋɨfni	ŋɨfnu	ŋafna	ŋafnaŋ	–	ŋafn

Rules:

$$[\quad] \rightarrow \begin{bmatrix} \text{+high} \\ \text{-low} \end{bmatrix} / \left[\text{ -sonorant } \right]\_$$

$$[\quad] \rightarrow \text{eː} / \_\left[\text{ -vowel } \right] \begin{bmatrix} \text{+continuant} \\ \text{-high} \end{bmatrix}$$

$$\begin{bmatrix} \text{+continuant} \\ \text{-high} \end{bmatrix} \rightarrow \text{o} / \left[\text{ -anterior } \right]\_\left[\text{ -vowel } \right]$$

$$\begin{bmatrix} \text{+continuant} \\ \text{-high} \end{bmatrix} \rightarrow \text{a} / \left[\text{ -coronal } \right]\_\left[\text{ +nasal } \right]$$

This problem was manually graded:

- V epenthesis: $\varnothing \rightarrow$ [αhigh, βlow, γbk] / [–syll, αhigh, βlow, γbk]C_C#. Missed by model.
- Height harmony: [-syl] $\rightarrow$ [+high] / _[+high]. Missed by model.
- a-raising: a $\rightarrow$ [+high] / [+high]+_ (raise a→ɨ in a suffix after a high vowel). Missed by model.
- V deletion: V $\rightarrow \varnothing$ / _V (ordered after a-raising). Missed by model.

All model rule predictions are spurious.

Lardil :

stem	stem+in	stem+ŋar	stem+uṛ	ÛR	UR
kentapal	kentapalin	kentapalŋar	kentapaluṛ	kentapal	kentapal
ketar	ketarin	ketarŋar	ketaruṛ	ketar	ketar
mijaṛ	mijaṛin	mijaṛŋar	mijaṛuṛ	mijaṛ	mijaṛ
jupur	jupurin	jupurŋar	jupuruṛ	jupur	jupur
taŋur	taŋurin	taŋurŋar	taŋuruṛ	taŋur	taŋur
jaraman	jaramanin	jaramanar	jaramankuṛ	jaraman	jaraman
maaṇ	maaṇin	maaṇar	maaṇkuṛ	maaṇ	maaṇ
pirŋen	pirŋenin	pirŋenar	pirŋenkuṛ	pirŋen	pirŋen
mela	melan	melaŋar	melaṛ	mela	mela
tawa	tawan	tawaŋar	tawaṛ	tawa	tawa
wanka	wankan	wankaŋar	wankaṛ	wanka	wanka
kuŋka	kuŋkan	kuŋkaŋar	kuŋkaṛ	kuŋka	kuŋka
tarŋka	tarŋkan	tarŋkaŋar	tarŋkaṛ	tarŋka	tarŋka
ŋuka	ŋukun	ŋukuŋar	ŋukuṛ	ŋuku	ŋuku
ŋuṛa	ŋuṛun	ŋuṛuŋar	ŋuṛuṛ	ŋuṛu	ŋuṛu
kaṭa	kaṭun	kaṭuŋar	kaṭuṛ	kaṭu	kaṭu
muṇa	muṇun	muṇuŋar	muṇuṛ	muṇu	muṇu
ŋawa	ŋawun	ŋawuŋar	ŋawuṛ	ŋawu	ŋawu
keṇṭe	keṇṭin	keṇṭiŋar	keṇṭiwuṛ	–	keṇṭi
tjimpe	tjimpin	tjimpiŋar	tjimpiwuṛ	–	tjimpi
ŋiṇe	ŋiṇin	ŋiṇiŋar	ŋiṇiwuṛ	–	ŋiṇi
pape	papin	papiŋar	papiwuṛ	–	papi
tjempe	tjempen	tjempeŋar	tjempeṛ	–	tjempe
wiṭe	wiṭen	wiṭeŋar	wiṭeṛ	–	wiṭe
waŋal	waŋalkin	waŋalkar	waŋalkuṛ	–	waŋalk
menjel	menjelkin	menjelkar	menjelkuṛ	–	menjelk
makar	makarkin	makarkar	makarkuṛ	–	makark
jalul	jalulun	jaluluŋar	jaluluṛ	–	jalulu
majar	majaran	majaraŋar	majaraṛ	–	majara
talkur	talkuran	talkuraŋar	talkuraṛ	–	talkura
wiwal	wiwalan	wiwalaŋar	wiwalaṛ	–	wiwala
karikar	karikarin	karikariŋar	karikariwuṛ	–	karikari
jilijil	jilijilin	jilijiliŋar	jilijiliwuṛ	–	jilijili
jukar	jukarpan	jukarpaŋar	jukarpaṛ	–	jukarpa
pulŋar	pulŋarpan	pulŋarpaŋar	pulŋarpaṛ	–	pulŋarpa
wulun	wulunkan	wulunkaŋar	wulunkaṛ	–	wulunka
wuṭal	wuṭaltjin	wuṭaltjiŋar	wuṭaltjiwur	–	wuṭaltji
kantukan	kantukantun	kantukantuŋar	kantukantuṛ	–	kantukantu
karwakar	karwakarwan	karwakarwaŋar	karwakarwaṛ	–	karwakarwa
turara	turaraŋin	turaraŋar	turaraŋkuṛ	–	turaraŋ
ŋalu	ŋalukin	ŋalukar	ŋalukuṛ	–	ŋaluk
kurka	kurkaŋin	kurkaŋar	kurkaŋkuṛ	–	kurkaŋ
taŋku	taŋkuŋin	taŋkuŋar	taŋkuŋkuṛ	–	taŋkuŋ
kurpuṛu	kurpuṛuŋin	kurpuṛuŋar	kurpuṛuŋkuṛ	–	kurpuṛuŋ
putu	putukan	putukaŋar	putukaṛ	–	putuka
maali	maalijan	maalijaŋar	maalijaṛ	–	maalija
tjiṇṭirpu	tjiṇṭirpuwan	tjiṇṭirpuwaŋar	tjiṇṭirpuwaṛ	–	tjiṇṭirpuwa
pukatji	pukatjijan	pukatjijaŋar	pukatjijaṛ	–	pukatjija
murkuni	murkuniman	murkunimaŋar	murkunimaṛ	–	murkunima
ŋawuŋa	ŋawuŋawun	ŋawuŋawuŋar	ŋawuŋawuṛ	–	ŋawuŋawa
tipiti	tipitipin	tipitipiŋar	tipitipiwuṛ	–	tipitipi
tapu	taputjin	taputjiŋar	taputjiwuṛ	–	taputji
muŋkumu	muŋkumuŋkun	muŋkumuŋkuŋar	muŋkumuŋkuṛ	–	muŋkumuŋku
tjumputju	tjumputjumpun	tjumputjumpuŋar	tjumputjumpuṛ	–	tjumputjumpu

Rules:

$$\begin{bmatrix} \text{+nasal} \end{bmatrix} \rightarrow \varnothing \; / \begin{bmatrix} \text{+nasal} \end{bmatrix} \_$$

$$\begin{bmatrix} \text{+vowel} \end{bmatrix} \rightarrow \varnothing \; / \begin{bmatrix} \text{+vowel} \end{bmatrix} \_ \begin{bmatrix} \text{+coronal} \end{bmatrix}$$

$$\begin{bmatrix} \text{+voice} \end{bmatrix} \rightarrow \text{a} \; / \begin{bmatrix} \quad \end{bmatrix} \begin{bmatrix} \text{-vowel} \end{bmatrix} \_ \#$$

$$\varnothing \rightarrow \text{k} \; / \begin{bmatrix} \text{+continuant} \\ \text{-high} \end{bmatrix} \begin{bmatrix} \text{+nasal} \end{bmatrix} \_ \begin{bmatrix} \quad \end{bmatrix} \begin{bmatrix} \text{-sonorant} \end{bmatrix}$$

Dutch :

stem+tə	ÛR	UR
klaptə	klap	klap
krabdə	krab	krab
rɛdə	rɛ	rɛd
vɪstə	vɪs	vɪs
razdə	raz	raz
zɛtə	zɛ	zɛt
maftə	maf	maf
klovdə	klov	klov
lɛɣdə	lɛɣ	lɛɣ
laxtə	lax	lax
rumdə	rum	rum
zundə	zun	zun
meŋdə	meŋ	meŋ
rurdə	rur	rur
rɔldə	rɔl	rɔl
ajdə	aj	aj
skidə	ski	ski

Rules:
[-vowel] $\rightarrow$ [+voice] / [-anterior] [+voice]_[+vowel]

Icelandic :

stem+ur	stem	stem+ri	stem	stem+ðum	stem	ÛR	UR
dagur	dag	–	–	–	–	dag	dag
staður	stað	–	–	stöðum	–	stað	stað
hestur	hest	–	–	–	–	hest	hest
bær	bæ	–	–	–	–	bæ	bæ
læknir	lækni	–	–	–	–	lækni	lækni
lifur	–	lifri	–	–	–	lif	lif
akur	–	agri	–	ökrum	–	–	ak
aldur	–	aldri	–	öldrum	–	–	ald
lüfur	lüf	–	lüfs	lüfjum	lüfja	–	lüfurj
bülur	bül	–	büls	büljum	bülja	–	bülj
söngur	söng	–	söngs	söngvum	söngva	–	söngv
barn	–	–	–	börnum	–	–	börn
baggi	–	–	–	böggull	–	–	bögg
jaki	–	–	–	jökull	–	–	jök
θagga	–	–	–	θögull	–	–	θög
kalla	–	–	–	köllum	–	–	köll

Rules:

$$[\quad] \rightarrow \text{ö} \;/\; \_\begin{bmatrix}\text{-sonorant}\\ \text{+voice}\end{bmatrix}[\text{ -vowel }]$$

$$[\text{ +voice }] \rightarrow \varnothing \;/\; [\text{ +vowel }]\_[\text{ -vowel }]$$

Anxiang :

stem	stem+iər	ÛR	UR
tie	tietiər	tiet	tiet
mian	mianmiər	mianm	mian
tai	taitər	tai	tai
pau	paupər	pau	pau
ke	kekər	ke	ke
fa	fafər	fa	fa
o	oər	o	o
ti	titiər	tit	ti
tin	tintiər	tint	tin
p^{h}u	p^{h}up^{h}ər	p^{h}u	p^{h}u
tx̯j	tx̯jtx̯jər	tx̯tx̯	tx̯j

Rules:

$$\begin{bmatrix}\text{-vowel}\end{bmatrix} \rightarrow \varnothing \;/\; \begin{bmatrix}\text{-back}\end{bmatrix}\_\#$$

$$\begin{bmatrix}\text{-back}\\ \text{+vowel}\end{bmatrix} \rightarrow \varnothing \;/\; \begin{bmatrix}\text{+vowel}\end{bmatrix}\_\begin{bmatrix}\text{+continuant}\\ \text{-high}\end{bmatrix}$$

$$\varnothing \rightarrow \begin{bmatrix}\text{-vowel}\end{bmatrix}_i \;/\; \#\begin{bmatrix}\text{-vowel}\end{bmatrix}_i\begin{bmatrix}\text{-anterior}\end{bmatrix}_0\_\begin{bmatrix}\text{-tense}\end{bmatrix}\begin{bmatrix}\text{+continuant}\\ \text{-high}\end{bmatrix}$$

$$\begin{bmatrix}\;\;\end{bmatrix} \rightarrow \text{j} \;/\; \text{x̯}\_$$

Supplementary References

[1] William F. Weigel. *Yowlumne in the Twentieth Century.* PhD thesis, University of California Berkeley, 2005.

[2] Armando Solar Lezama. *Program Synthesis By Sketching.* PhD thesis, University of California Berkeley, 2008.

[3] Daniel Perelman, Sumit Gulwani, Dan Grossman, and Peter Provost. Test-driven synthesis. In *ACM Sigplan Notices*, volume 49, pages 408–418. ACM, 2014.

[4] David Odden. *Introducing phonology.* Cambridge university press, 2005.
