# Supplementary material for: Synthesizing theories of human language with Bayesian program induction
Source: Nat Commun. 2022 Aug 30;13:5024. doi: 10.1038/s41467-022-32012-w (PMC9427767; doi:10.1038/s41467-022-32012-w)
Supplement: Supplementary file 3 — Reporting Summary [file 41467_2022_32012_MOESM3_ESM.pdf]

nature portfolio

Corresponding author(s): Kevin Ellis

Last updated by author(s): May 24, 2022

Reporting Summary

Nature Portfolio wishes to improve the reproducibility of the work that we publish. This form provides structure for consistency and transparency in reporting. For further information on Nature Portfolio policies, see our Editorial Policies and the Editorial Policy Checklist.

Statistics

For all statistical analyses, confirm that the following items are present in the figure legend, table legend, main text, or Methods section.

n/a	Confirmed	
☐	☒	The exact sample size (*n*) for each experimental group/condition, given as a discrete number and unit of measurement
☒	☐	A statement on whether measurements were taken from distinct samples or whether the same sample was measured repeatedly
☐	☒	The statistical test(s) used AND whether they are one- or two-sided<br>*Only common tests should be described solely by name; describe more complex techniques in the Methods section.*
☒	☐	A description of all covariates tested
☒	☐	A description of any assumptions or corrections, such as tests of normality and adjustment for multiple comparisons
☐	☒	A full description of the statistical parameters including central tendency (e.g. means) or other basic estimates (e.g. regression coefficient) AND variation (e.g. standard deviation) or associated estimates of uncertainty (e.g. confidence intervals)
☐	☒	For null hypothesis testing, the test statistic (e.g. *F*, *t*, *r*) with confidence intervals, effect sizes, degrees of freedom and *P* value noted<br>*Give P values as exact values whenever suitable.*
☐	☒	For Bayesian analysis, information on the choice of priors and Markov chain Monte Carlo settings
☒	☐	For hierarchical and complex designs, identification of the appropriate level for tests and full reporting of outcomes
☐	☒	Estimates of effect sizes (e.g. Cohen's *d*, Pearson's *r*), indicating how they were calculated

Our web collection on statistics for biologists contains articles on many of the points above.

Software and code

Policy information about availability of computer code

Data collection	Sketch version 1.7.5
Data analysis	Python 2.7

For manuscripts utilizing custom algorithms or software that are central to the research but not yet described in published literature, software must be made available to editors and reviewers. We strongly encourage code deposition in a community repository (e.g. GitHub). See the Nature Portfolio guidelines for submitting code & software for further information.

Data

Policy information about availability of data

All manuscripts must include a data availability statement. This statement should provide the following information, where applicable:

- Accession codes, unique identifiers, or web links for publicly available datasets
- A description of any restrictions on data availability
- For clinical datasets or third party data, please ensure that the statement adheres to our policy

The language data used and generated in this study have been deposited in GitHub at https://github.com/ellisk42/bpl_phonology along with the accompanying source code (DOI 0.5281/zenodo.6578329) under the GPLv3 license. The numerical data generated in this study are provided in the Source Data file.

Field-specific reporting

Please select the one below that is the best fit for your research. If you are not sure, read the appropriate sections before making your selection.

☐ Life sciences ☒ Behavioural & social sciences ☐ Ecological, evolutionary & environmental sciences

For a reference copy of the document with all sections, see nature.com/documents/nr-reporting-summary-flat.pdf

Behavioural & social sciences study design

All studies must disclose on these points even when the disclosure is negative.

Study description	We performed a quantitative study using computational simulations on linguistic corpora and randomly generated artificial grammar learning stimuli. We did not collect or use any data from human participants.
Research sample	We collected and transcribed 70 linguistic corpora from textbook exercises and examples. They were selected to exemplify a range of linguistic phenomena and difficulty levels. The three textbooks we used were Introducing Phonology (David Odden), A Workbook in Phonology (Iggy Roca and Wyn Johnson), and Problem Book in Phonology (Morris Halle and George Clements). We also created synthetic data for artificial grammar learning simulations. The synthetic data comprised 3 syllable words, with each syllable having one consonant and one vowel, with the consonant drawn uniformly at random from a set of 21 possible consonants and the vowel drawn uniformly at random from a set of 11 vowels.
Sampling strategy	We randomly sampled 15 problems to manually evaluate (grade) the predicted phonological rules. We chose this sample size because manually grading is a labor intensive process, involving consultation with a professional phonologist, and this sample of 15 sufficed to establish a statistically significant correlation between rule accuracy and lexicon accuracy (Figure 5C). For each artificial grammar learning stimulus, we randomly sampled 30 test words drawn as described under "Research sample", and structured them into 15 word-pairs for log-odds ratio computations. This sample size was chosen because it showed that the distribution of log-odds ratios was low variance.
Data collection	We did not collect any data from human participants.
Timing	We collected data from Introducing Phonology from September 18 2016 until September 16 2017. We collected data from A Workbook in Phonology and Problem Book in Phonology from January 24, 2019 to February 25, 2019
Data exclusions	We excluded phonology problems requiring autosegmental tiered representations. These are presented as an advanced topic in the last chapter of Odden's Introducing Phonology, and do not conform to the Sound Patterns of English rule format. There are 3 such problems, and we excluded them from the outset of data set creation.
Non-participation	n/a, because we did not have human participants
Randomization	n/a, because we did not have human participants

Reporting for specific materials, systems and methods

We require information from authors about some types of materials, experimental systems and methods used in many studies. Here, indicate whether each material, system or method listed is relevant to your study. If you are not sure if a list item applies to your research, read the appropriate section before selecting a response.

Materials & experimental systems

n/a	Involved in the study
☒	☐ Antibodies
☒	☐ Eukaryotic cell lines
☒	☐ Palaeontology and archaeology
☒	☐ Animals and other organisms
☒	☐ Human research participants
☒	☐ Clinical data
☒	☐ Dual use research of concern

Methods

n/a	Involved in the study
☒	☐ ChIP-seq
☒	☐ Flow cytometry
☒	☐ MRI-based neuroimaging
